# MiRNA-340-5p mediates the functional and infiltrative promotion of tumor-infiltrating CD8^+^ T lymphocytes in human diffuse large B cell lymphoma

**DOI:** 10.1186/s13046-020-01752-2

**Published:** 2020-11-10

**Authors:** Yangyang Xu, Zhenchuan Liu, Lixin Lv, Ping Li, Bing Xiu, Wenbin Qian, Aibin Liang

**Affiliations:** 1 Department of Hematology, Tongji Hospital, Tongji University School of Medicine, Shanghai, 200065 China; 2 Department of Thoracic and Cardiovascular Surgery, Tongji Hospital, Tongji University School of Medicine, Shanghai, 200065 China; 3 Department of Hematology, the Second Affiliated Hospital, College of Medicine, Zhejiang University, Hangzhou, 310009 China

**Keywords:** DLBCL, T-TILs, CD8^+^ T cells, MiR-340-5p

## Abstract

**Background:**

CD8^+^ tumor-infiltrating T lymphocytes (T-TILs) in the tumor microenvironment (TME) play an important role in tumor development, and miRNAs regulate tumor cell interactions with the microenvironment. T-TIL-based tumor immunotherapy provides a promising treatment strategy in diffuse large B-cell lymphoma (DLBCL). MiRNAs tend to be attractive targets for novel antitumor interventions.

**Methods:**

Weighted gene coexpression network analysis (WGCNA), CIBERSORT analysis and Cox regression analysis were used to identify CD8^+^ T-TIL-related miRNAs. RT-PCR, western blotting, immunohistochemistry (IHC), in situ hybridization (ISH), luciferase reporter assay, coimmunoprecipitation and ubiquitination analyses were used to detect miRNA, mRNA and protein expression and their combination. The viability and function of CD8^+^ T cells after stimulation were evaluated by enzyme-linked immunosorbent assay (ELISA), cytotoxicity assay, functional avidity assessment, flow cytometry and Cell Counting Kit-8 (CCK-8) assay. DLBCL cell lines, primary cells and a murine xenograft model established with A20 cell injection were used as in vitro and in vivo experimental models.

**Results:**

MiR-340-5p was positively correlated with CD8^+^ T-TILs in DLBCL patients, and KMT5A was a direct target gene of miR-340-5p. CD8^+^ T-cell function was significantly enhanced by miR-340-5p mimics both in vitro and in vivo, which was reversed by KMT5A overexpression. We demonstrated that COP1/CD73 was involved in the downstream mechanism of the miR-340-5p/KMT5A axis involving ubiquitination. In vivo, we validated an improved CD8^+^ T-TIL infiltration rate and tumor suppression with miR-340-5p treatment. Furthermore, miR-340-5p directly regulated the biological activity of DLBCL cells without CD8^+^ T-cell participation.

**Conclusions:**

MiR-340-5p promoted CD8^+^ T-TIL infiltration and antitumor function by regulating KMT5A and COP1 and further activating CD73 ubiquitination. MiR-340-5p is potentially a novel target for DLBCL immunotherapy.

**Supplementary Information:**

The online version contains supplementary material available at 10.1186/s13046-020-01752-2.

## Background

MiRNAs are a class of small endogenous noncoding RNAs that regulate multiple genes by binding to the 3′ untranslated region (3′ UTR) of their mRNA post-transcriptionally [1]. With an oncogenic role, several key dysregulations of miRNAs can lead to the generation and progression of many tumor cells, including the development of hematopoietic malignancies [2–4]. Several models have been constructed in which miRNAs contribute to the robustness of cell fates and functional responses in the hematopoietic system [5–8]. Diffuse large B-cell lymphoma (DLBCL) is an aggressive B-cell malignancy and is typically fatal in patients not cured after initial therapy. Although rituximab emerged as the hallmark of immunotherapeutic success, standard therapy (R-CHOP) can offer long-term remission to a minority of patients [9]. DLBCL is characterized by its intertumoral heterogeneity, consisting of therapeutically different molecular abnormalities. Recently, miRNA signatures provided novel information for specific drug resistance and clinical outcome in chemoimmunotherapy-treated DLBCL patients, and miRNA signatures also regulate tumor cell interactions with their microenvironment [10–12]. Given their pivotal role in the understanding of DLBCL biology, miRNAs have been considered emerging cancer biomarkers and potential candidates for therapeutic targets.

During neoplastic initiation and development, cellular and molecular signatures have also been reported to be dysregulated in cells and the matrix from their tumor microenvironment (TME) [13]. It has been increasingly noticed that tumor cells bear a broad range of dependence on interactions with a plethora of immune cells and stromal matrix in the TME [14]. Among different types of T lymphocytes, CD8^+^ T cells conduct immune surveillance to eliminate cancer cells and develop malignancies as the major immune cells. Tumor-infiltrating T lymphocytes (T-TILs) isolated from non-Hodgkin’s B-cell lymphomas genetically differed from those in reactive lymph nodes, and similar changes could be triggered by coculturing normal T cells with lymphoma cells [15]. This result suggests that T-TILs are affected by either direct cellular contact or a paracrine pattern. Therefore, it remains to be answered whether or not extracellular elements and CD8^+^ T-TILs in the TME shift lymphoma sensitivity to cytotoxic T lymphocyte (CTL)-mediated elimination of malignant cells.

In this work, with a bioinformatics screening, we identified miR-340-5p and demonstrated its biological effects on the TME in DLBCL cells. We further revealed KMT5A, also known as SET domain-containing protein 8 (SETD8), as a target gene of miR-340-5p, which probably influences CD8^+^ T cells in the TME of DLBCL by the COP1/CD73 axis. We determined the promotive effects of miR-340-5p on CD8^+^ T lymphocyte infiltration and elucidated the underlying mechanisms utilizing in vitro and in vivo models.

## Materials and methods

### Data collection, preprocessing and analysis

**Fig. 1:**
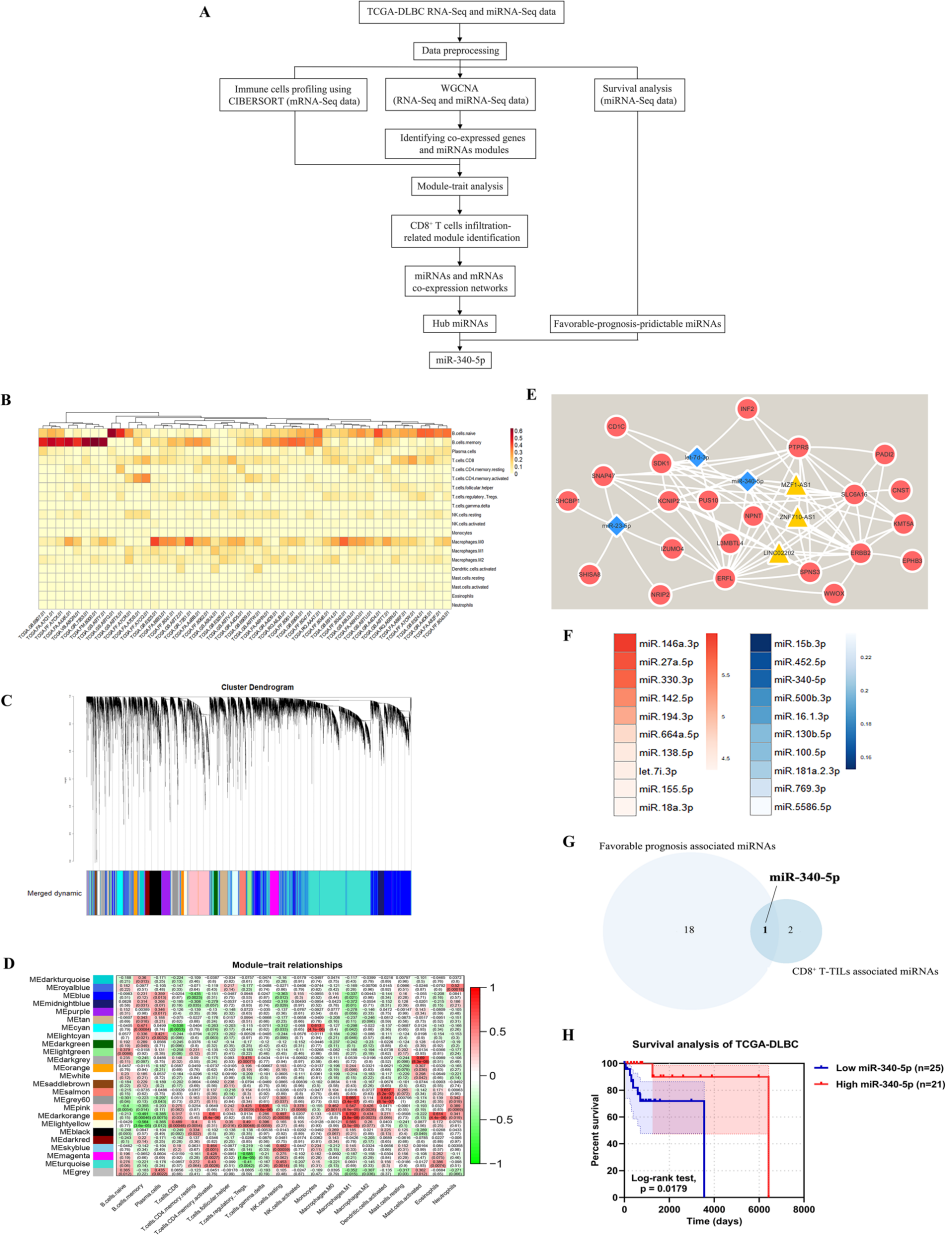
Bioinformatics identification of CD8^+^ T-TIL-related miR-340-5p in DLBCL. **a** Flow chart of data preprocessing and analysis. TCGA, The Cancer Genome Atlas; DLBCL, diffuse large B-cell lymphoma; WGCNA, weighted gene coexpression network analysis. **b** The heatmap summarizes the proportions of immune cell subsets (CIBERSORT *p* value < 0.05). **c** Clustering dendrograms of genomic signatures. In all, 25 modules were identified by the dynamic tree cut method, and each module was symbolized by a color. **d** Gene and miRNA module–trait associations. Each row corresponded to a module eigengene, and each column corresponded to a tumor-infiltrating immune cell subtype. The corresponding correlation and q-value are presented from top to bottom in each cell. The cells were color-coded by correlation according to the color legend. **e** MiRNA-gene from the MEcyan module interaction network. MiRNAs are shown in blue, and genes are shown in red. The lines between miRNAs and genes indicate coexpression relationships among them. **f** MiRNAs ranked in the top 10 with HR > 1 (left panel, red) or HR < 1 (right panel, blue) according to Cox regression analysis. **g** Intersection of hub miRNAs in WGCNA and survival-associated miRNAs (HR < 1). **h** Survival analysis of miR-340-5p in DLBCL patients

A workflow of the bioinformatics analysis in this study is shown in Fig. 1a. The RNA-sequencing (RNA-seq) and miRNA isoform expression quantification (miRNA-seq) data in the “TCGA-DLBC” project were collected from The Cancer Genome Atlas (TCGA; http://cancergenome.nih.gov/) on December 4, 2018. Raw counts of RNA/miRNA expression data were normalized by trimmed means of M values (TMM) implemented in edgeR and then transformed by voom in limma. Low-expression genes and miRNAs were filtered out; only genes and miRNAs with counts per million reads (cpm) > 1 in more than half of the samples were retained. The RNA-sequencing (RNA-seq) and miRNA isoform expression quantification (miRNA-seq) data of DLBCL contained 48 and 47 DLBCL samples, respectively. After outlier samples and samples with incomplete clinical information were screened out, 46 DLBCL samples remained for subsequent bioinformatics analysis.

Weighted gene coexpression network analysis (WGCNA) was performed in the TCGA-DLBC cohort, and a miRNA-gene interaction network was visualized using Cytoscape v3.4.0. Cox regression and survival analysis were carried out after sample classification according to the mean of miRNA or gene expression level. The RNA-seq data from these samples were subjected to immune cell infiltration profiling using CIBERSORT [16]. We used the LM22 leukocyte gene signature matrix, which includes 547 genes distinguishing 22 hematopoietic cell phenotypes and acquired tumor-infiltrating immune cell profiling with a CIBERSORT *p* value < 0.05.

### Human subjects

DLBCL patients enrolled in this study provided informed consent, and specimens were collected at diagnosis biopsy from Shanghai Tongji Hospital Affiliated to Tongji University. None of the subjects received anticancer treatment before biopsy. The protocol was approved by the Institutional Review Board of Center for Medicine, Shanghai Tongji Hospital. All studies were conducted in accordance with the Declaration of Helsinki. Peripheral blood mononuclear cells (PBMCs) were isolated from heparinized whole blood by Ficoll/Hypaque density gradient centrifugation (Solarbio, China) followed by CD8^+^ T-cell-positive selection using CD8 MicroBeads (Miltenyi, Germany).

### Cell culture

The human DLBCL cell lines (LY-1, LY-7) were obtained from the Cell Bank of the Chinese Academy of Sciences (China). The murine B lymphoma cell line A20 was purchased from American Type Culture Collection (ATCC) (USA). LY-1 and LY-7 cells were cultured in Iscove’s modified Dulbecco’s medium (IMDM, Gibco, USA), and A20 cells were cultured in RPMI 1640 medium (Gibco, USA) supplemented with 10% fetal bovine serum (FBS) (HyClone, USA) and 1% penicillin/streptomycin (HyClone, USA) in a humidified atmosphere of 5% CO_2_ at 37 °C. For LY-7 and A20 cells, 0.05 mM β-mercaptoethanol was added to the culture medium. Primary CD8^+^ T cells were cultured in RPMI 1640 medium supplemented with 10% FBS, 1% L-glutamine, 1% penicillin/streptomycin and 200 IU/mL IL-2. To stimulate CD8^+^ T cells, 2 μg/mL of the CMV peptide pool was used for the stimulation of 250,000 cells per well. In direct coculture, CD8^+^ T cells were harvested and dispensed into 96-well plates according to various effector:target ratios, which were described in the corresponding experiments. LY-1 or LY-7 cells were then added into each CD8^+^ T cell-containing well at a density of 20,000 cells per well. When the cocultures in ELISA, cytotoxic assay and functional avidity assay were described, CD8^+^ T cells were preincubated with anti-CD3/anti-CD28 Dynabeads (ThermoFisher, USA) (bead: T-cell ratio = 1:1) overnight and stimulated to achieve substantial expansion. For indirect coculture, tumor cells were seeded into Transwell chambers with a 0.4 μm aperture membrane and then transferred to a 24-well plate seeded with CD8^+^ T cells in advance, and the supernatant was collected for designed experiments.

### Transfection

Oligonucleotides for miR-340-5p inhibition and forced expression were purchased from GenePharma (China). The specific siRNA, recombinant plasmids KMT5A-OE, FLAG-CD73, HA-COP1, 6x-His-Ub, pLVX-shKMT5A-PURO, pLVX-shCOP1-PURO and their corresponding negative controls were generated and purchased from KeLei Biological Technology (China). The lentivirus was packaged with Δ89 and VSVG helper plasmids, and DLBCL cells were transfected with polybrene, followed by centrifugation at 2500×g for 90 min at 37 °C. Oligonucleotides, siRNA and plasmids were transfected using Lipofectamine 3000 (Invitrogen, USA) following the manufacturer’s protocols. Cells were subjected to experiments after 24 h of infection. The sequences of shRNA, miRNA mimics and miRNA inhibitors are available in the Supplemental Information (Tables 1 and 2).

### RT-PCR

Total RNA was extracted using TRIzol reagent (Invitrogen, USA) by phenol–chloroform precipitation. MiRNAs were reverse transcribed individually by using miRNA-specific reverse transcription primers and the One Step miRNA cDNA Synthesis Kit (HaiGene Bio Inc., China), while total RNA was reverse transcribed into cDNA using the PrimeScript RT Reagent Kit with gDNA Eraser (Takara, Japan). Real-time quantitative RT-PCR was conducted using SYBR Green technology (Takara, Japan) and ABI QuantStudio 6 (USA). U6 and GAPDH were used as endogenous controls for PCR analysis of miRNAs and mRNAs, respectively. Each experiment was run in triplicate. Data were analyzed according to the 2^-ΔΔCt^ method.

### Western blotting

Cells were rinsed 3 times with precooled phosphate-buffered saline (PBS) and lysed by RIPA and phenylmethylsulfonyl fluoride (PMSF). Total protein was harvested in 1× sodium dodecyl sulfate (SDS) loading buffer after centrifugation and denaturation. Protein samples were separated by sodium dodecyl sulfate polyacrylamide gel electrophoresis (SDS-PAGE) and electrotransferred to polyvinylidene fluoride membranes (Millipore, USA) with an electrophoresis system (Bio-Rad, CA). The membranes were incubated with horseradish peroxidase (HRP)-conjugated anti-rabbit or anti-mouse secondary antibodies for 1 h at room temperature followed by immunoreactive band detection and analysis.

### IHC and ISH

KMT5A and CD8 expression was determined by immunohistochemistry (IHC) for all 40 DLBCL samples. Formalin-fixed paraffin-embedded (FFPE) tissue sections were deparaffinized and dehydrated in xylene and graded ethanol solutions. After deparaffinization, antigen recovery was performed in an autoclave using citrate buffer (pH 6.0) for 15 min, and the slides were then cooled at room temperature and washed in PBS. Slides were incubated with 3% H_2_O_2_ and goat serum in the proper order as described in the kit manual (Elabscience, China). Primary antibodies were incubated overnight at 4 °C. 3,3′-Diaminobenzidine (DAB) and hematoxylin staining were performed the next day. ISH was performed to detect miR-340-5p expression using an ISH kit (BOSTER, China). Experimental procedures followed the manufacturer’s instructions as previously described [17]. Briefly, FFPE samples were stained with DAB and hematoxylin after dehydration and sealing. Oligo (5′ Digoxin-AATCAGTCTCATTGCTTTATAA-3′) was used as the miR-340-5p ISH probe.

For quantification in human specimens and murine models, at least two investigators trained in lymphoma blindly assessed the immunohistochemical staining and achieved a final consensus. Slides were first scanned at low magnification (10x magnification), and 10 high magnification fields (400x magnification) were assessed. For scoring gene and miRNA expression, the intensity of staining was classified into 0 (no expression), 1 (weak expression), 2 (moderate expression) and 3 (high expression), and the percentage of positive cells was categorized as 1 (positive cells ≤25%), 2 (25% < positive cells ≤50%), 3 (50% < positive cells ≤75%) and 4 (positive tumor cells > 75%) [18, 19]. The histochemical score (H-score) was achieved by multiplying the staining intensity and the percentage of positive cells and ranged from 0 to 300. The mean of H-score was considered the cut-off point [18]. Any cell with CD8-positive staining was counted as CD8^+^ T-TIL. For DLBCL patient samples, the intratumoral area was selected for CD8^+^ T-TIL evaluation. CD8^+^ T-TILs were counted manually in each high-power field and scored as follows: 0 (none), 1 (1–2 CD8^+^ T-TILs), 2 (3–19 CD8^+^ T-TILs), and 3 (≥20 CD8^+^ T-TILs) [20, 21].

### Antibodies and reagents

The following primary antibodies were used: anti-tubulin (Abcam, ab210797), anti-KMT5A (Abcam, ab111691), anti-CD8 (Abcam, ab17147), anti-CD8 (Abcam, ab217344), anti-CD3 (Abcam, ab16669), anti-ubiquitin (CST, #43124), anti-CD69 (Abcam, ab54217), anti-FLAG (Abcam, ab205606), anti-HA (Abcam, ab236632), anti-6X His (Abcam, ab213204), anti-CD73 (Abcam, ab54217), anti-CD73 (Abcam, ab54217), anti-COP1 (Abcam, ab56400), anti-MKRN1 (Abcam, ab72054), anti-MDM2 (BOSTER, BA3612–2), and anti-Ki-67 (CST, #12202), anti-CD69 (BOSTER, A00529–2), anti-IFN-γ (Abcam, ab231036), anti-IL-2 (Abcam, ab92381), anti-TNF-α (Abcam, ab270264), anti-perforin (Abcam, ab47225), anti-Granzyme B (BOSTER, A00353), anti-perforin (Abcam, ab16074). The following secondary antibodies were used: goat anti-mouse (CST) and goat anti-rabbit (CST). MG132 and cycloheximide (CHX) were purchased from CST (USA). The CMV peptide pool stimulating CD8^+^ T cells was obtained from Mabtech (Sweden).

### Luciferase reporter assay for the 3′ UTR study

**Fig. 2:**
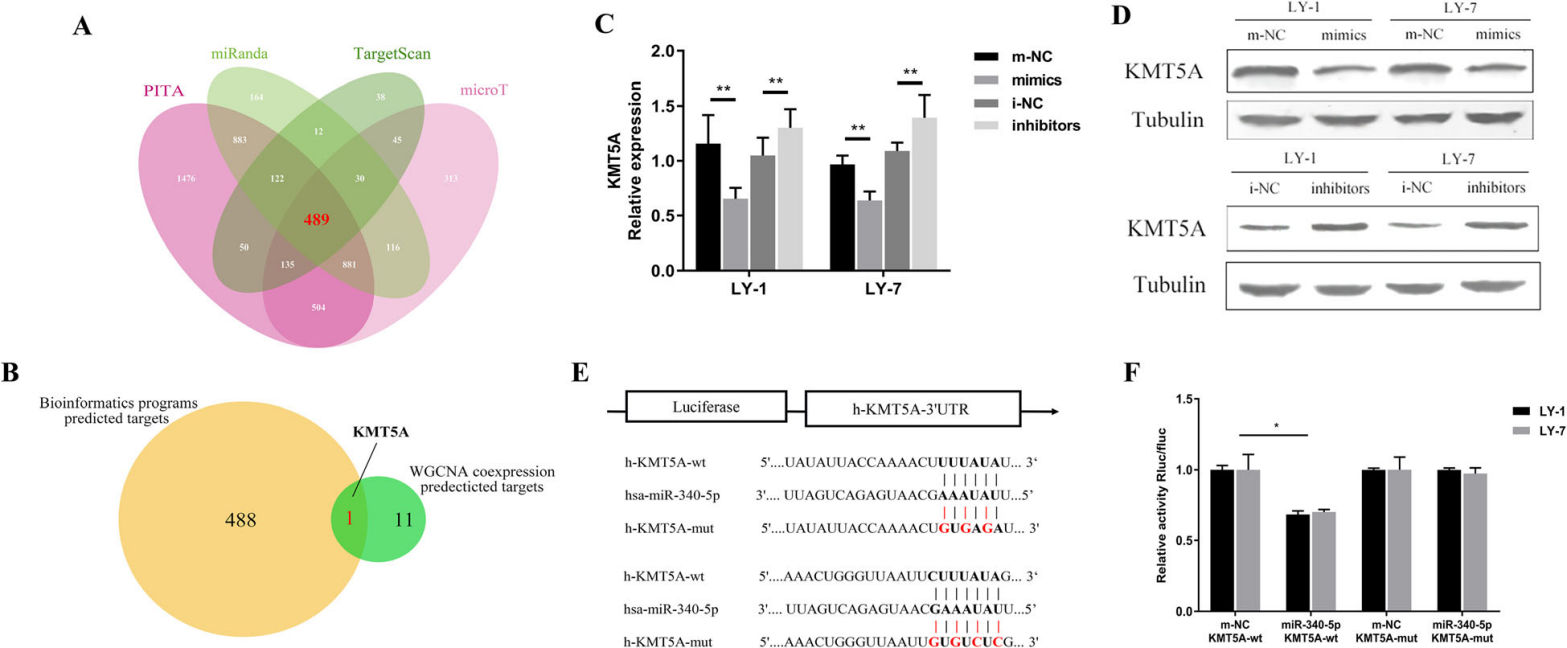
KMT5A is a direct target of miR-340-5p. **a** Intersection of miR-340-5p predicted targets from 4 prediction algorithms (PITA, miRanda, TargetScan, and microT), and 489 genes were selected. **b** Of the 489 genes, KMT5A was the only gene presented in both bioinformatics-predicted targets and miR-340-5p coexpression analysis. **c**-**d** KMT5A expression regulated by miR-340-5p was confirmed by RT-PCR (**c**) and western blotting (**d**). **e** The putative miR-340-5p-binding sites within the 3′ UTR of KMT5A mRNA. Relevant mutations are shown. **f** Reporter plasmids containing either the wild-type (wt) or mutated (mut) 3′ UTR of KMT5A were cotransfected with miR-340-5p. Luciferase activity was significantly decreased in wt, while mut remained unchanged. Asterisks (* and **) indicate a significant difference. *, P < 0.05; **, P < 0.01

Luciferase reporter plasmids carrying the wild-type (wt) or mutated (mut) KMT5A 3′ UTR were constructed and purchased from Hanbio (China). Two predicted miR-340-5p-binding sites were simultaneously mutated and linked into plasmid carriers. The reporter plasmid was transfected into DLBCL cells along with miR-340-5p mimics or its negative control using Lipofectamine 3000 (Invitrogen, USA). Cells were lysed 48 h after transfection, and luciferase activity was measured using the Dual-Luciferase Reporter Assay System (Promega, USA). The sequences of KMT5A-3′-UTR-wt and KMT5A-3′-UTR-mut are shown in Fig. 2e.

### Elisa

CD8^+^ T cells were cocultured with LY-1 or LY-7 cells (effector:target ratio, E:T = 30:1), and the cell culture supernatant was centrifuged for 20 min at 1000×g at 2–8 °C. The supernatant was collected to carry out the assay. The supernatant was diluted according to preliminary experiments and the detection range for IFN-γ, TNF-α, IL-2. Working solutions of the standard or samples were added to plates and prepared according to the manufacturer’s instructions. The optical density (OD) value of each well was determined simultaneously with a microplate reader set to 450 nm. All of the reagents were obtained from Elabscience (China).

### LDH assay

The cytotoxicity assays were conducted using the CytoTox 96 NonRadioactive Cytotoxicity Assay Kit (Promega, USA). According to the protocol, CD8^+^ T cells were cocultured with DLBCL cell lines LY-1 or LY-7 in a 96-well U-bottom plate at various E:T ratios of 3:1, 10:1, and 30:1 for 4 h. Subsequently, 50 μl of supernatant per well was collected to detect lactate dehydrogenase (LDH) release in a microplate imaging system at an absorbance of 490 nm. As controls, the spontaneous release of LDH was evaluated by incubating T cells or target cells alone, and the maximum release of LDH was assessed by incubating target cells in 0.1% Triton X-100. The results of specific LDH release were calculated as follows: percent specific release = [(experimental OD − effector spontaneous OD − target spontaneous OD)/(target maximum OD − target spontaneous OD)] × 100%.

### Functional avidity assessment

The functional avidity of antigen-specific stimulated CD8^+^ T cells was assessed by limited peptide dilutions and IFN-γ production. The concentration that gives a half-maximal response (EC_50_) of the peptide to mobilize half of the maximal CD8^+^ T-cell response was used as the measurement of antigen sensitivity, which was independent of the magnitude of the CD8^+^ T-cell response with saturated antigen [22]. The EC_50_ of the peptide required to achieve a half-maximal IFN-γ response was determined as previously described [23]. Peptide stimulation was performed as described in the *Cell culture*.

### Flow cytometry

Cells were washed with cold PBS and suspended in PBS containing 10% FBS and 1% sodium azide at a concentration of 1 × 10^6^ cells/mL. Cell staining was performed with antibodies for 30 min, followed by cell washing and flow cytometry analysis. For apoptosis analysis, apoptotic cells were detected using the Annexin V-fluorescein isothiocyanate (FITC)/propidium iodide (PI) Kit (KeyGEN BioTECH, China) according to the manufacturer’s instructions. Briefly, cells with the indicated treatment or transfection were collected and stained with FITC and PI in the dark for 15 min and subjected to flow cytometry. Samples were analyzed using FACSVerse (BD Biosciences) and FlowJo software (Tree Star, USA).

### CCK-8 assay

Cells transfected with the indicated lentivirus or oligonucleotides were seeded in 96-well plates for cell viability analysis using Cell Counting Kit-8 (CCK-8; Dojindo, Japan). CCK-8 assays were performed with six replicates, and OD values at 450 nm were measured using a microplate imaging system.

### Coimmunoprecipitation and ubiquitination analysis

For endogenous ubiquitination detection, ubiquitination analysis was performed as previously described [24]. Briefly, cells were harvested after the indicated treatments and lysed with modified RIPA buffer. After sonication, the lysates were boiled, diluted and centrifuged. The supernatant was subjected to immunoprecipitation with specific antibodies. CD73 ubiquitination and protein detection were determined by western blotting. For the nickel pull-down assay, cell lysates were prepared with lysis buffer. The lysates were sonicated for 30 s, followed by incubation with 50 mL Ni-NTA-agarose (Qiagen, CA) for 4 h at room temperature. The beads were washed with a specific washing buffer, boiled with 2 × SDS loading buffer containing 200 mM imidazole and subjected to western blotting.

### Murine model

Female adult BALB/c mice (4 weeks old, obtained from Shanghai Laboratory Animal Center, Shanghai, China) were injected with 1 × 10^7^ A20 cells into the right flank [12]. All mouse experiments were conducted with approval from the Experimental Animal Committee of Shanghai Tongji Hospital. For miR-340-5p, agomirs (GenePharma, China) were delivered on three consecutive days, and intratumoral injections of agomirs and their controls were injected at doses of 30 mg/kg per injection [25]. α,β-Methylene adenosine-5′-diphosphate (APCP, Sigma-Aldrich, USA) was injected intratumorally at a daily dose of 20 mg/kg for 1 week followed by twice weekly [26, 27]. Treatments started 1 week after the tumor challenge, and volume measurements started after the tumor reached approximately 0.5 cm × 0.5 cm on the surface (Day 0). Tumor volumes were calculated as 0.5 × a (length) × b (width)^2^. For flow cytometry, tumor tissues were dissected into approximately 2 mm^3^ fragments, plated in 24-well plate wells individually and digested using an enzyme mix including DNAse, collagenase, and hyaluronidase. For IHC, tumor tissues were dissected into FFPE or formalin/paraformaldehyde (PFA)-fixed paraffin-embedded sections and subjected to IHC staining and scoring, as described in *Immunohistochemistry*. Treatments did not cause a significant reduction in body weight (Supplemental Figure 4).

### Statistical analysis

Statistical analysis was performed using GraphPad Prism software. All data are presented as the mean ± SD of at least three independent experiments. We evaluated the data with Student’s t test. Differences between nonparametric data were analyzed by the Mann-Whitney U test, and multigroup comparisons were performed using one-way analysis of variance (ANOVA). A *p* value < 0.05 was considered significant. In all charts, the mean and standard error are presented.

## Results

### Bioinformatics analysis unveiled that miR-340-5p was related to CD8^+^ T-TILs in DLBCL

To obtain critical genes and miRNAs related to CD8^+^ T-TILs in DLBCL, we analyzed the TCGA-DLBC cohort using survival analysis, CIBERSORT and WGCNA, as the workflow described in Fig. 1a. We first profiled the immune cell infiltration rate (Fig. 1b) and identified 25 modules of WGCNA (Fig. 1c). To identify genes and miRNAs associated with tumor-infiltrating immune cells, we carried out module–trait relationship analysis (Fig. 1d). Among various immune cells, CD8^+^ T cells exerted the final elimination of tumor cells as CTLs and played a more important role in tumor immunotherapy. Therefore, although multiple modules were detected to be related to immune cell infiltration, we focused on the correlations between genomic features and the CD8^+^ T-TIL infiltration rate. We found that the MEcyan module possessed a significant correlation and the highest absolute value of r (*r* = − 0.538, q = 0.0053) (Fig. 1d**)**.

Based on the genes and miRNAs in the MEcyan module, we constructed a coexpression network and detected three hub miRNAs (miR-340-5p, let-7d-3p, and miR-23-5p) (Fig. 1e). Since the hub miRNAs were selected from the module associated with patients’ CD8^+^ T-TIL infiltration fraction, it was of great significance to evaluate their potential in prognosis. The miRNA-seq data and survival information of 46 previously described DLBCL patients were analyzed using Cox regression analysis with a *p* value < 0.05. According to the hazard ratios (HRs), the miRNAs ranked in the top 10 in the HR > 1 and HR < 1 groups are shown in Fig. 1f. We focused on miRNAs associated with promotive effects on CD8^+^ T-TILs and took the overlap of the three hub miRNAs and the favorable survival-predictable miRNA set (HR < 1), and only miR-340-5p remained (Fig. 1g). Moreover, patients with higher miR-340-5p levels achieved longer survival times (Fig. 1).

### KMT5A was detected to be a functional target of miR-340-5p

MiRNAs play an important role in tumor cells by regulating the expression of their target genes. To identify target genes of miR-340-5p, we computationally nominated genes that might be regulated by miR-340-5p. We integrated the miR-340-5p target prediction results of four bioinformatics programs, PITA (https://genie.weizmann.ac.il/pubs/mir07/mir07_data.html), TargetScan (http://www.targetscan.org/), miRanda (http://www.microrna.org/microrna/home.do) and DIANA-microT (https://omictools.com/diana-microt-cds-tool). Overall, 489 putative target genes were predicted to overlap with the results of these prediction algorithms (Fig. 2a). We then determined the overlapping genes revealed by WGCNA coexpression analysis of miR-340-5p and the bioinformatics programs that predicted genes, as elaborated above. Only KMT5A remained in the intersection and gave us a clue to the molecular mechanism of miR-340-5p functions in DLBCL (Fig. 2b).

To confirm the bioinformatics-predicted target gene of miR-340-5p, western blotting and RT-PCR were performed. The results revealed that the KMT5A expression level was significantly downregulated by the forced expression of miR-340-5p compared with its negative control, the m-NC-transfected cells, and inversely upregulated by the miR-340-5p inhibitor (Fig. 2c-d). Furthermore, we conducted a luciferase reporter assay to determine whether KMT5A was a direct target of miR-340-5p in DLBCL cells, according to binding sites predicted by TargetScan (http://www.targetscan.org/vert_72/) (Fig. 2e). The wt potential target region sequence of the KMT5A 3′-untranslated region (3′ UTR) or a mutant sequence carrying two putative miR-340-5p binding sites (MUT1 and MUT2 3′ UTR) was cloned into luciferase reporter vectors. Our results demonstrated that miR-340-5p significantly decreased the luciferase activity of the KMT5A wt 3′ UTR construct with the cotransfection of miR-340-5p mimics, while the inhibited luciferase activity was abolished with mutations in the potential binding sites (Fig. 2f). These results suggested that KMT5A was a novel target of miR-340-5p.

### MiR-340-5p improved the function of CD8^+^ T lymphocytes cocultured with DLBCL cells either directly or indirectly

**Fig. 3:**
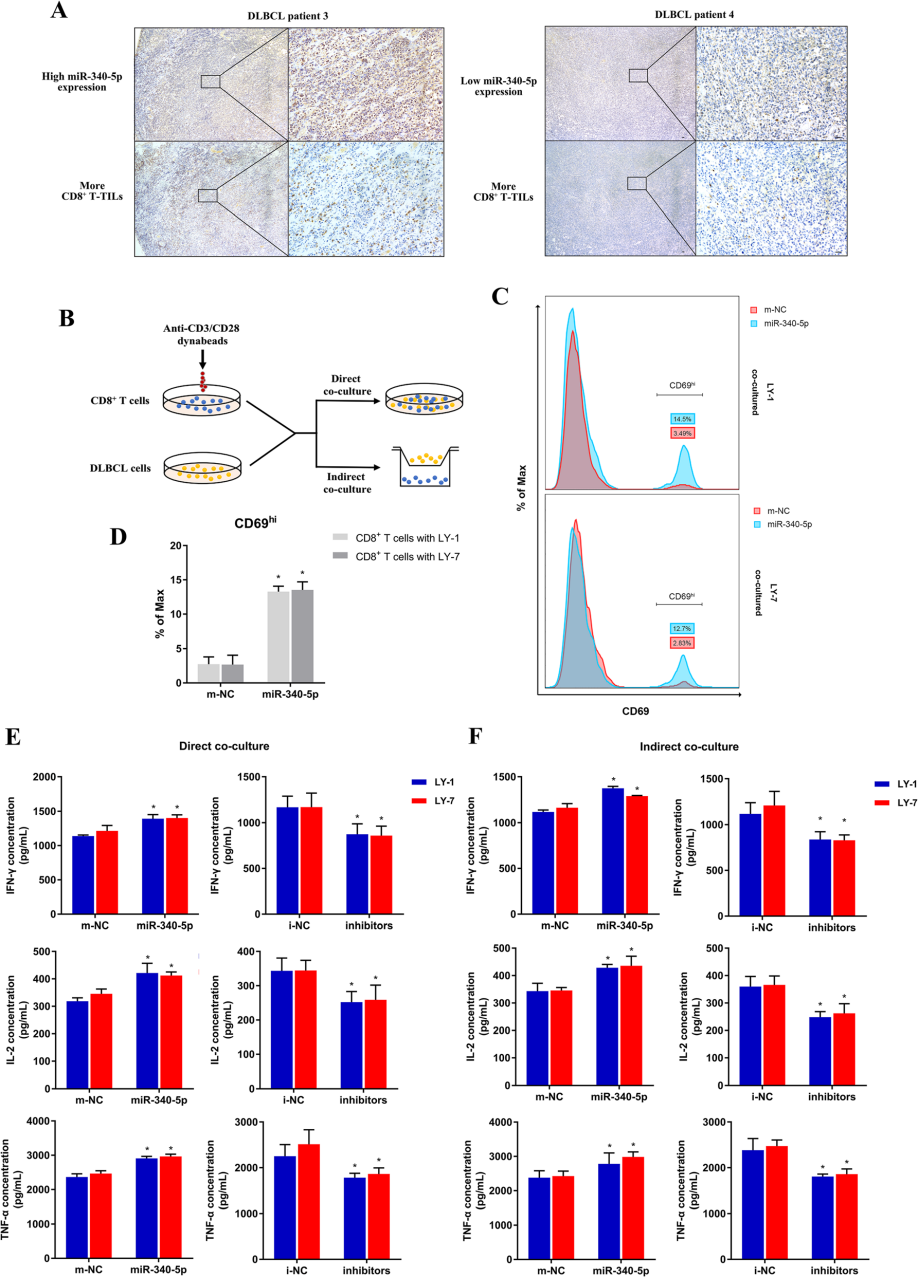
CD8^+^ T-cell immune function is facilitated by miR-340-5p. **a** Representative ISH images of the association between miR-340-5p expression and CD8^+^ T-TILs in DLBCL patients. Magnification of the microscope: left column, 40×; right column, 200×. **b** Schema for coculturing CD8^+^ T cells and DLBCL cells. **c-d** The representative proportion (**c**) and cumulative data (**d**) of CD69^hi^ cells in CD8^+^ T cells. **e-f** Cytokine production assays (IFN-γ, TNF-α, and IL-2) were performed for CD8^+^ T cells directly (**e**) or indirectly (**f**) cocultured with DLBCL cell lines transfected with m-NC or miR-340-5p. Asterisk (*) indicates a significant difference. *, P < 0.05

To investigate whether miR-340-5p played a role in the regulation of DLBCL-induced CD8^+^ T cell suppression, we first verified the bioinformatics-implied correlation between miR-340-5p level and CD8^+^ T-TILs in DLBCL patients. We observed higher CD8^+^ T-TIL infiltration fractions in patients with high miR-340-5p expression, and vice versa (Fig. 3a). After ISH scoring and the Mann-Whitney U test, we demonstrated a positive correlation between miR-340-5p and CD8^+^ T-TILs. To further explore the effects of miR-340-5p on CD8^+^ T cell function, we cocultured primary CD8^+^ T cells with DLBCL cells. Primary CD8^+^ T cells were previously activated with anti-CD3/anti-CD28 beads, and Transwell chambers were utilized in indirect coculturing manner (Fig. 3b). DLBCL cells were transfected with miR-340-5p mimics, miR-340-5p inhibitors, or their corresponding negative control oligonucleotides m-NC or i-NC. We evaluated the expression of the early activation marker CD69 in CD8^+^ T cells directly cocultured with LY-1 or LY-7 cells [28]. The number of T cells with high CD69 expression (CD69^hi^) increased in cells cocultured with miR-340-5p mimic-transfected cells (Fig. 3c-d). Because there were various immune-reactive and immune-suppressive molecules on the cell membrane, we cocultured DLBCL cells with CD8^+^ T cells in either a direct or indirect way to investigate whether cell-cell contact took place in the mechanism by which miR-340-5p affected CD8^+^ T cells. We assessed the functional properties of CD8^+^ T cells by measuring cytokine production (i.e., IFN-γ, TNF-α, and IL-2) using enzyme-linked immunosorbent assay (ELISA). Regardless of having direct cell-cell contact, CD8^+^ T cells produced more IFN-γ, TNF-α, and IL-2 when exposed to miR-340-5p-overexpressing DLBCL cells, while miR-340-5p suppression inhibited cytokine production (Fig. 3e-f). These results confirmed that miR-340-5p in DLBCL cells facilitated CD8^+^ T cell function and implied that miR-340-5p modulated soluble immune-regulatory molecules produced by DLBCL cells.

We also investigated whether miR-340-5p had a direct impact on CD8^+^ T cells independent of DLBCL cells. After forced or inhibited expression of miR-340-5p, cell viability, T cell activation and cytokine production were evaluated by CCK-8, flow cytometry and ELISA (Supplemental Figure 1). We did not confirm a significant difference after miR-340-5p interference.

### KMT5A regulated CD8^+^ T-cell function as a downstream target of miR-340-5p

**Fig. 4:**
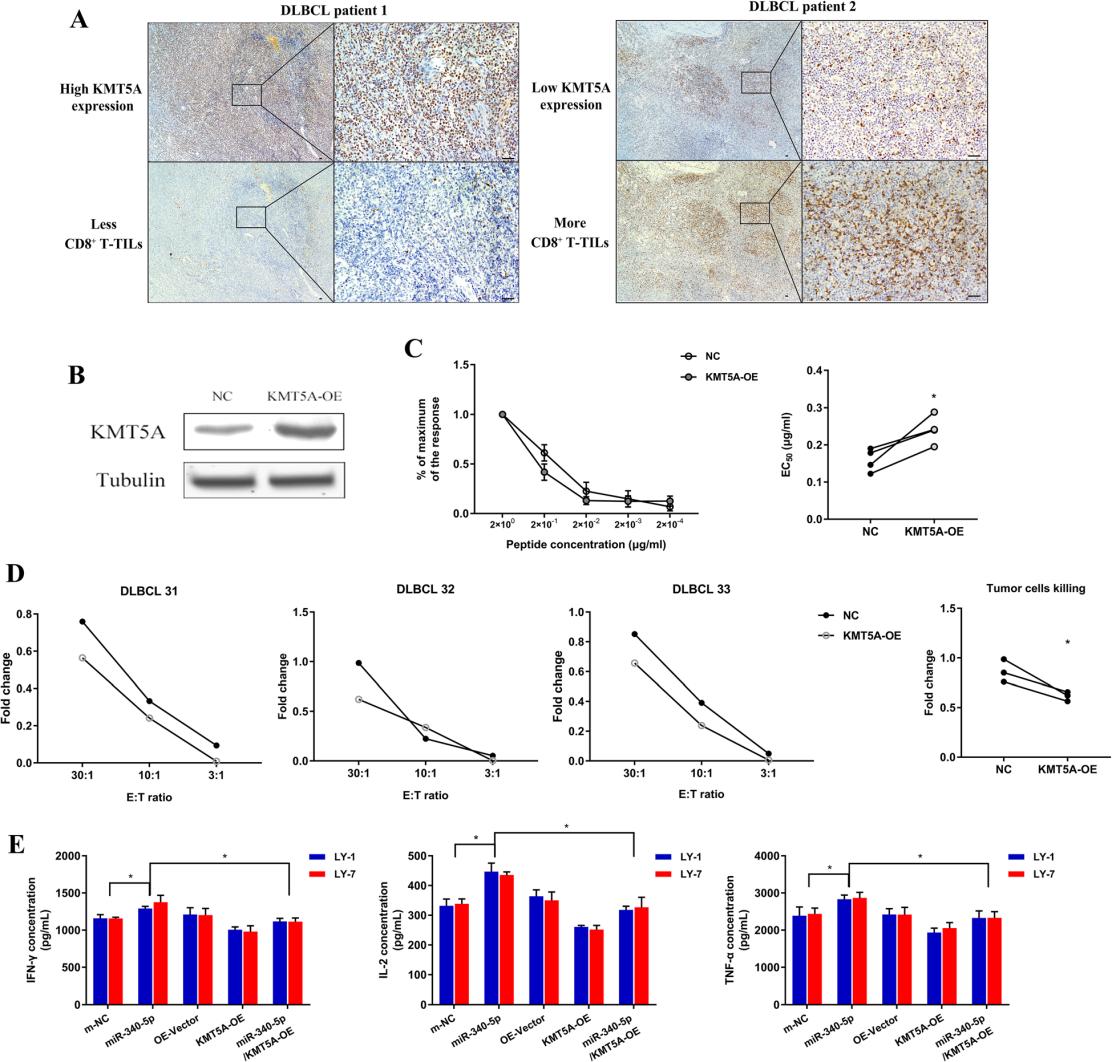
The miR-340-5p/KMT5A axis regulates CD8^+^ T cells. **a** Representative IHC images of the association between KMT5A expression and CD8^+^ T-TILs. Magnification of the microscope: left column, 40×; right column, 200×. **b** KMT5A overexpression was confirmed by western blotting. **c** Representative example and cumulative data of the virus-specific CD8^+^ T-cell functional avidity. **d** Each graph represents cytotoxicity curves for one DLBCL patient. CD8^+^ T-cell cytotoxicity was quantified with KMT5A-overexpressing or negative control LY-1 cells. Cumulative data of the fold change in cytotoxicity are shown. **e** KMT5A overexpression significantly reversed the miR-340-5p-induced increase in cytokine production in cocultured CD8^+^ T cells. Asterisk (*) indicates a significant difference. *, P < 0.05

We previously confirmed that KMT5A was a direct target of miR-340-5p. To further identify the correlation of KMT5A and CD8^+^ T-TILs, we next used 30 tumor samples from DLBCL patients collected at diagnosis. The correlation between KMT5A and CD8 protein expression was analyzed after IHC staining. KMT5A was negatively correlated with CD8^+^ T-TIL infiltration in DLBCL specimens (Wilcoxon rank-sum test, *p* value < 0.05) (Fig. 4a).

Furthermore, we previously observed that forced expression of miR-340-5p in DLBCL cells enhanced the function of cocultured T cells, and we aimed to demonstrate the function of KMT5A in CD8^+^ T cell regulation mediated by miR-340-5p. We then overexpressed KMT5A in DLBCL cells and confirmed its expression by western blotting (Fig. 4b). Considering that CD8^+^ T-cell functional avidity was demonstrated to be related to superior control of tumor growth [29], we then cultured CMV peptide pool-stimulated CD8^+^ T cells with KMT5A-overexpressing or negative control LY-1 cells. We evaluated the functional avidity of CD8^+^ T cells in peptide concentration gradients and observed that in the KMT5A-overexpression group, it was significantly downregulated (Fig. 4c). These data indicated that KMT5A facilitated the immunosuppressive ability of DLBCL cells. Moreover, the cytotoxicity assay was performed using KMT5A-overexpressing or negative control LY-1 cells as the target cells and CD8^+^ T cells as effector cells. Then, an LDH assay was performed, and the negative control DLBCL cells triggered a relatively stronger cytotoxicity in an E:T ratio-dependent manner, while T cells cocultured with KMT5A-overexpressing DLBCL cells displayed a relatively lower capacity for tumor cell lysis (Fig. 4d).

**Fig. 5:**
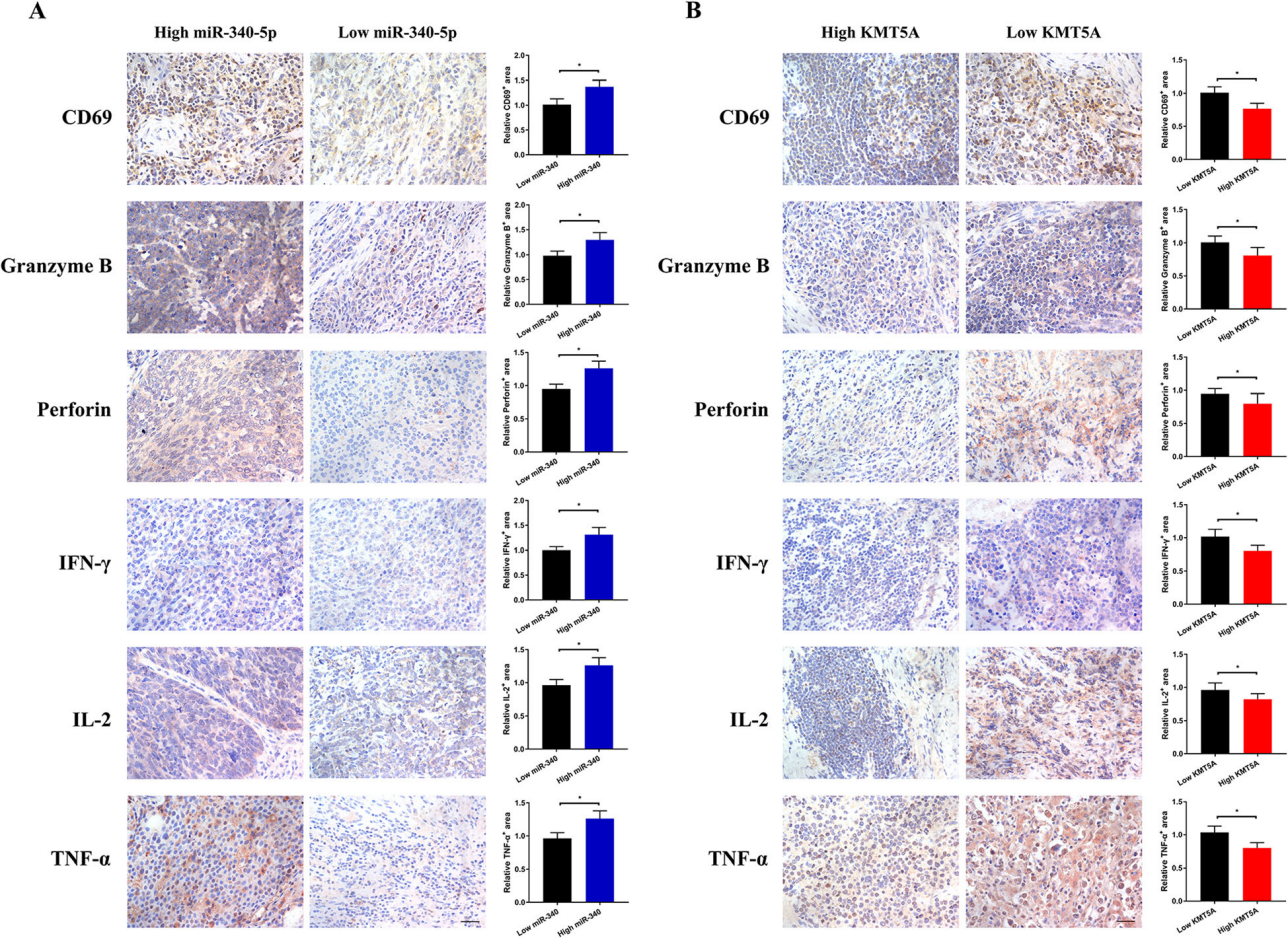
Functional molecules of CD8^+^ T-TILs differed in DLBCL specimens classified according to miR-340-5p or KMT5A expression. **a-b** Representative and cumulative IHC data of CD69, IFN-γ, TNF-α, IL-2, granzyme B and perforin in DLBCL samples, classified according to the mean of H-score of miR-340-5p (**a**) or KMT5A (**b**) expression. Asterisk (*) indicates a significant difference. *, *P* < 0.05

Regarding the suppressive effect of KMT5A on CD8^+^ T cells, we performed rescue experiments. CD8^+^ T-cell function analysis using cocultured DLBCL cells indicated that the overexpression of KMT5A in DLBCL cells could significantly eliminate the improvement of cytokine production of cocultured T cells caused by miR-340-5p (Fig. 4e). We further evaluated the expression levels of CD69 and functional molecules produced by CD8^+^ T-TILs, including IFN-γ, TNF-α, IL-2, granzyme B and perforin, in previously described DLBCL patients. Consistently, significantly higher levels of molecules representing CD8^+^ T-TIL activation and function were observed in DLBCL patients with higher miR-340-5p or lower KMT5A expression, while lower miR-340-5p or higher KMT5A expression implied fewer functional molecules of CD8^+^ T-TILs (Fig. 5a-b). Considering the above findings, we presumed that the miR-340-5p/KMT5A axis regulated CD8^+^ T-TIL infiltration in DLBCL.

### Silencing KMT5A downregulated CD73 via enhanced ubiquitination

**Fig. 6:**
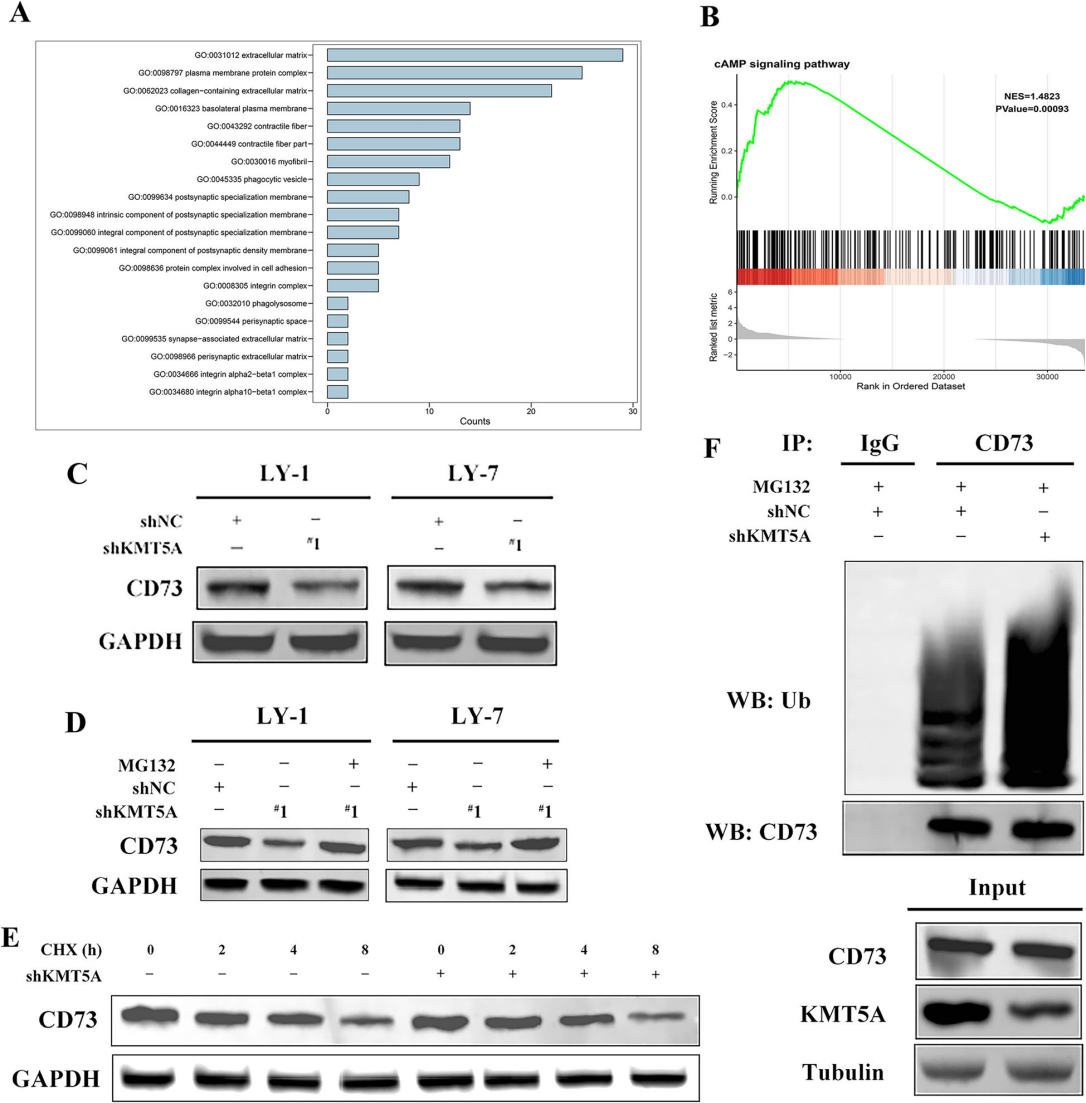
CD73 ubiquitination is increased by KMT5A knockdown. **a** GO enrichment analysis. **b** cAMP signaling pathway enrichment in GSEA. **c** western blot analysis of CD73 in KMT5A knockdown DLBCL cells. **d** MG132 rescued KMT5A silencing-induced CD73 downregulation. DLBCL cells transfected with shKMT5A were treated with MG132 or DMSO (control) for 24 h. **e** Endogenous CD73 stability in LY-1 cells stably overexpressing KMT5A. DLBCL cells were treated with CHX for various time points. **f** KMT5A silencing promoted CD73 ubiquitination. KMT5A was stably silenced in DLBCL cells. Cells were treated with MG132 for 24 h

We observed that the miR-340-5p/KMT5A axis could regulate CD8^+^ T cell function via the noncell contact pathway. To explore the specific underlying mechanism, we performed GSEA (Gene Set Enrichment Analysis) and GO (Gene Ontology) analysis using RNA-sequence data of cancer cells with KMT5A inhibition (GSE81626) [30] in GEO database (https://www.ncbi.nlm.nih.gov/gds). Top 20 GO terms in “cellular component” were shown in Fig. 6a. We found that “extracellular matrix” and “plasma membrane protein complex” were ranked top 2 in GO enrichment. Consistently, the gene set “cAMP signaling pathway” was revealed in GSEA top enriched sets (Table 1, Fig. 6b). Extracellular ATP metabolism and adenosine are known to produce important signaling molecules of the immune system. During this process, CD39 and CD73 degrade ATP, ADP, and AMP to adenosine and are critical mediators of adenosine accumulation in the TME [31]. Therefore, we then evaluated the changes in the expression of CD39 and CD73 after KMT5A knockdown, which was confirmed by western blotting (Supplemental Figure 2A). The protein level of CD73 was downregulated, which was assessed by western blotting, while its mRNA level remained unchanged in the RT-PCR assay (Fig. 6c, Supplemental Figure 2B), whereas neither the protein nor the mRNA level of CD39 changed (Supplemental Figure 2C-D). This observation demonstrated that CD73 was probably degraded post-transcriptionally as a result of KMT5A knockdown. Moreover, MG132, a proteasome inhibitor, restored CD73 expression, while a CHX chase experiment showed that KMT5A overexpression increased CD73 degradation (Fig. 6d-e). The data implied a ubiquitination and proteasome degradation mechanism in regard to how KMT5A affects CD73. Indeed, ubiquitination analysis revealed that KMT5A silencing increased CD73 ubiquitination in LY-1 cells (Fig. 6f). However, rather than a ubiquitination enzyme, KMT5A is the only known specific lysine methyltransferase that monomethylates histone H4 at lysine 20 (H4K20me1) [32]. The methyltransferase activity of KMT5A plays crucial roles in various cell biological processes, including transcriptional regulation and cell metabolism [33]. Therefore, our results indicated that ubiquitination enzymes were involved in the KMT5A-mediated regulation of CD73.

**Table 1:** Top 20 gene sets enriched after KMT5A inhibition in GSEA

ID	Description	Set Size
hsa05168	Herpes simplex virus 1 infection	486
hsa04740	Olfactory transduction	433
hsa04024	cAMP signaling pathway	213
hsa05203	Viral carcinogenesis	200
hsa05169	Epstein-Barr virus infection	197
hsa05034	Alcoholism	180
hsa04360	Axon guidance	179
hsa03013	RNA transport	162
hsa04218	Cellular senescence	156
hsa03010	Ribosome	151
hsa03040	Spliceosome	131
hsa05322	Systemic lupus erythematosus	129
hsa04110	Cell cycle	124
hsa01200	Carbon metabolism	115
hsa05012	Parkinson disease	114
hsa04061	Viral protein interaction with cytokine and cytokine receptor	99
hsa03008	Ribosome biogenesis in eukaryotes	94
hsa04640	Hematopoietic cell lineage	93
hsa04146	Peroxisome	82

### COP1 was downregulated by KMT5A and mediated CD73 ubiquitination and degradation

**Fig. 7:**
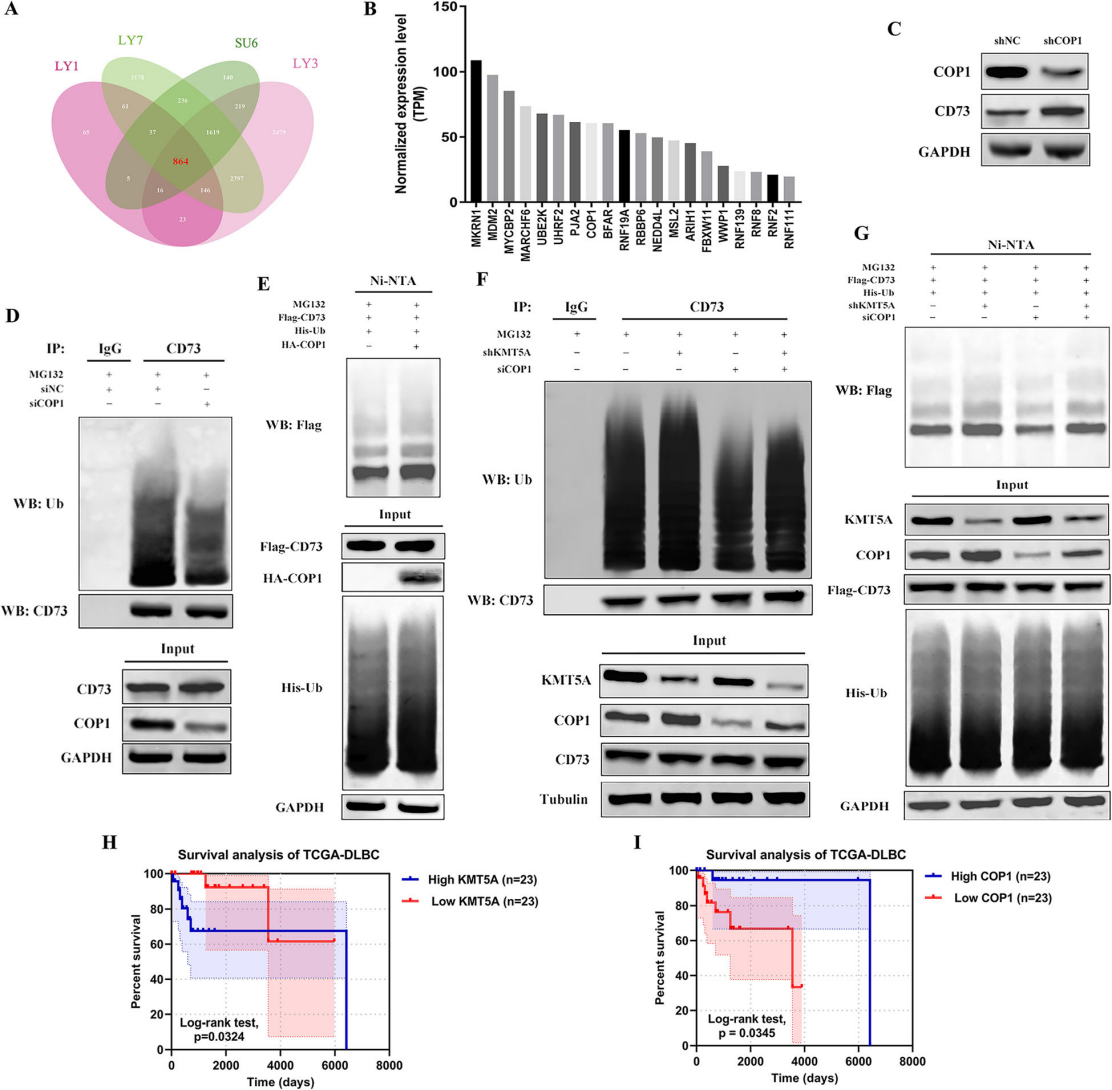
COP1 was required for KMT5A silencing-induced CD73 ubiquitination. **a** By analyzing ChIP-seq data of DLBCL, 864 selected genes were revealed to interact with H4K20 me1. **b** Expression levels of 20 E3 ligases selected by combining CHIP-seq analysis and GO annotations of E3 ligase. **c** COP1 downregulation restored CD73 expression. **d** COP1 knockdown DLBCL cells were treated with MG132 and subjected to coimmunoprecipitation and western blot analyses. **e** DLBCL cells were transfected with various plasmids, as indicated, and subjected to ubiquitination analysis. **f** DLBCL cells were transfected as indicated and subjected to coimmunoprecipitation and western blot analyses. **g** DLBCL cells were transfected as indicated and subjected to ubiquitination analysis. **h**-**i** Survival analysis of DLBCL patients classified according to KMT5A (**h**) or COP1 (**i**) expression

To further demonstrate the specific role and target of KMT5A in DLBCL immunosuppression and CD73 regulation, we analyzed previously published H4K20me1 chromatin immunoprecipitation sequencing (ChIP-seq) data of DLBCL cell lines: OCI-LY1, OCI-LY3, OCI-LY7, and SU-DHL-6 (GSE96492, GSE96374, GSE96350, and GSE96226, respectively) [34], using the “chip seeker” package in the R program; a total of 864 genes were selected in the overlap of these four cell lines’ data (Fig. 7a). Then, we integrated 864 genes in the overlap with Gene Ontology (GO) annotations of ubiquitin protein ligase activity (GO:0061630). Twenty H4K20me1-interacting genes were unveiled in the intersection (UBE2K, MDM2, RBBP6, RNF2, RNF8, PJA2, WWP1, RNF139, MARCHF6, MYCBP2, FBXW11, NEDD4L, MKRN1, ARIH1, RNF19A, BFAR, RNF111, MSL2, COP1, and UHRF2). Referring to the “TCGA-DLBC” project, normalized gene expression (transcripts per million reads, TPM) of these above described genes is shown in Fig. 7b. We selected several genes exerting E3 ligase activity and playing vital roles in cancers, with relatively higher expression levels among the 20 genes. The western blotting results showed that CD73 was upregulated with COP1 knockdown, whereas CD73 remained unchanged with the silencing of other genes (Fig. 7c, Supplemental Figure 3). To further validate CD73 ubiquitination in DLBCL cells, we performed ubiquitination analysis, and COP1 knockdown reduced CD73 ubiquitination in LY-1 cells (Fig. 7d). We then cotransfected CD73, COP1 and ubiquitin in LY-1 cells. The results revealed that COP1 robustly promoted CD73 ubiquitination (Fig. 7e).

In the reciprocal experiment, COP1 knockdown significantly reversed the enhanced ubiquitination of CD73 caused by KMT5A silencing (Fig. 7f). We then cotransfected CD73 and ubiquitin into LY-1 cells with shKMT5A and/or siCOP1. The data also showed a partial rescue effect of siCOP1 on CD73 ubiquitination after KMT5A was knocked down (Fig. 7g). Consistently, our analysis of the TCGA dataset revealed that COP1 was associated with better overall survival in DLBCL patients (*P* < 0.05), while KMT5A predicted poorer prognosis (Fig. 7h-i). These results demonstrated that KMT5A regulated CD73 ubiquitination by suppressing COP1.

### The miR-340-5p/KMT5A axis induced a more immunoreactive TME in vivo

**Fig. 8:**
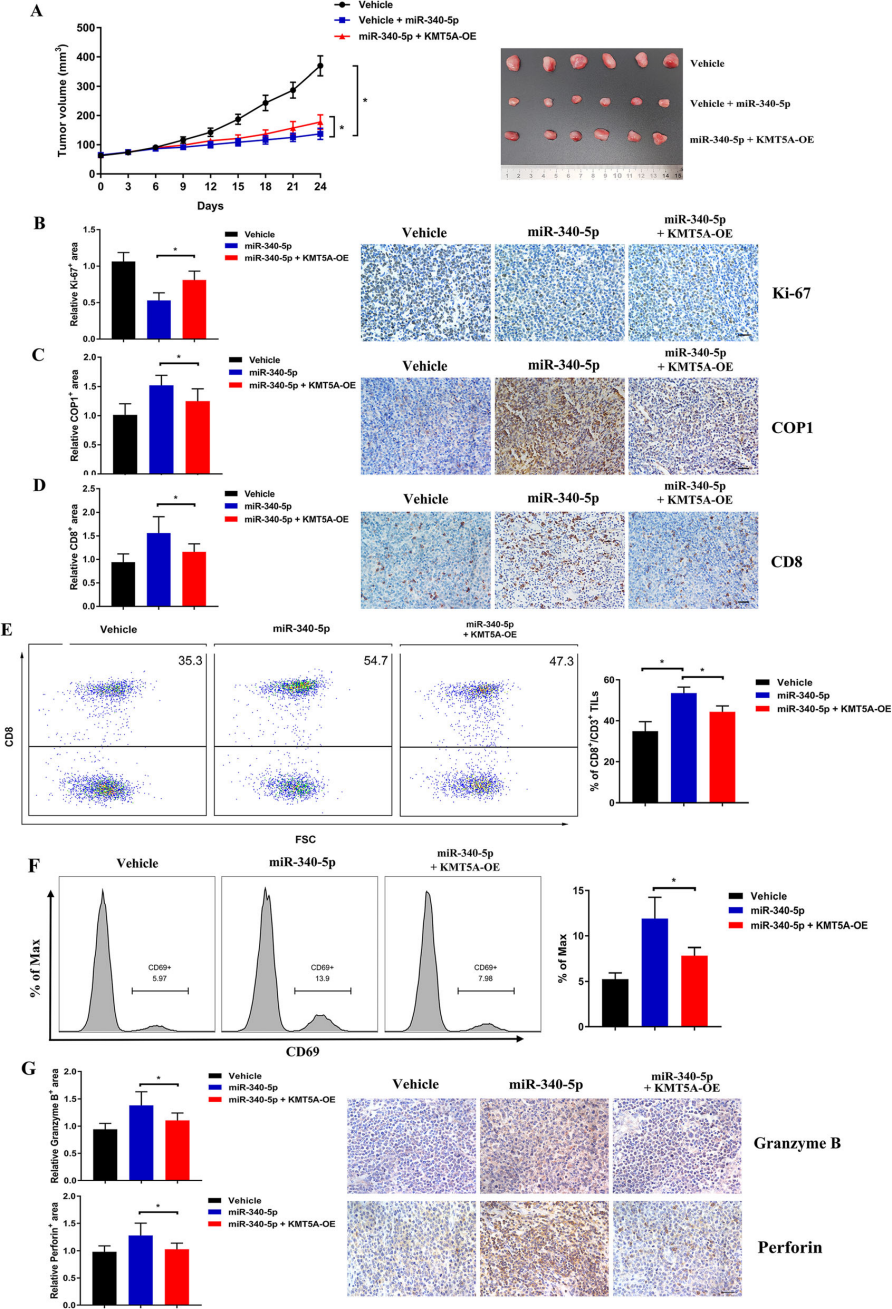
MiR-340-5p suppressed tumor growth by enhancing CD8^+^ T-TILs in vivo. **a** KMT5A significantly abrogated the lower tumor growth rate caused by miR-340-5p. Statistical tests were performed using tumor volume data on Day 24. **b**-**d** IHC analysis of Ki-67 (**b**), COP1 (**c**) and CD8 (**d**) expression in tumors, as indicated. The CD8^+^ T-TIL percentage was increased under miR-340-5p treatment and significantly reversed by overexpressing KMT5A, as shown by IHC analysis. **e** Representative flow cytometry data and cumulative data for CD8^+^ T-TILs isolated from murine model tumors. In all experiments, cells were first gated on the CD3^+^ population and then on the CD8^+^ population. **f** Representative flow cytometry data and cumulative data for CD69-positive CD8^+^ T-TILs. **g** IHC analysis of granzyme B and perforin. Asterisk (*) indicates a significant difference. *, P < 0.05

To further validate our results conducted in vitro, a murine xenograft model was established with subcutaneous injection of A20 cells stably transfected with the KMT5A-overexpression lentivirus or its negative control, with or without the forced expression of miR-340-5p [12, 35]. Compared with the negative control (vehicle), miR-340-5p exerted antitumor activity, which was significantly reversed by KMT5A overexpression (Fig. 8a). The differences in tumor growth were consistent with Ki-67 expression (Fig. 8b). COP1 expression increased with miR-340-5p and decreased when combined with KMT5A overexpression (Fig. 8c). We therefore investigated tumor-infiltrating leukocytes by performing IHC and flow cytometry analyses. The number of CD8^+^ T-TILs increased with miR-340-5p delivery and then decreased when KMT5A-OE was present (Fig. 8d). We further analyzed the CD8^+^ T-TIL proportion in CD3^+^ T-TILs by flow cytometry, and its miRNA-dependent increase was significantly reversed by overexpressing KMT5A (Fig. 8e). Functionally, miR-340-5p increased the CD69-positive CD8^+^ T-TIL proportion, and the effect was reversed by KMT5A overexpression (Fig. 8f). Cytotoxic molecules (i.e., granzyme B, perforin) was also increased in miR-340-5p-overexpressing tumor cells and significantly reversed by KMT5A-OE (Fig. 8g).

### The tumor-promoting effects of COP1 knockdown were overcome in vivo by a CD73 inhibitor

**Fig. 9:**
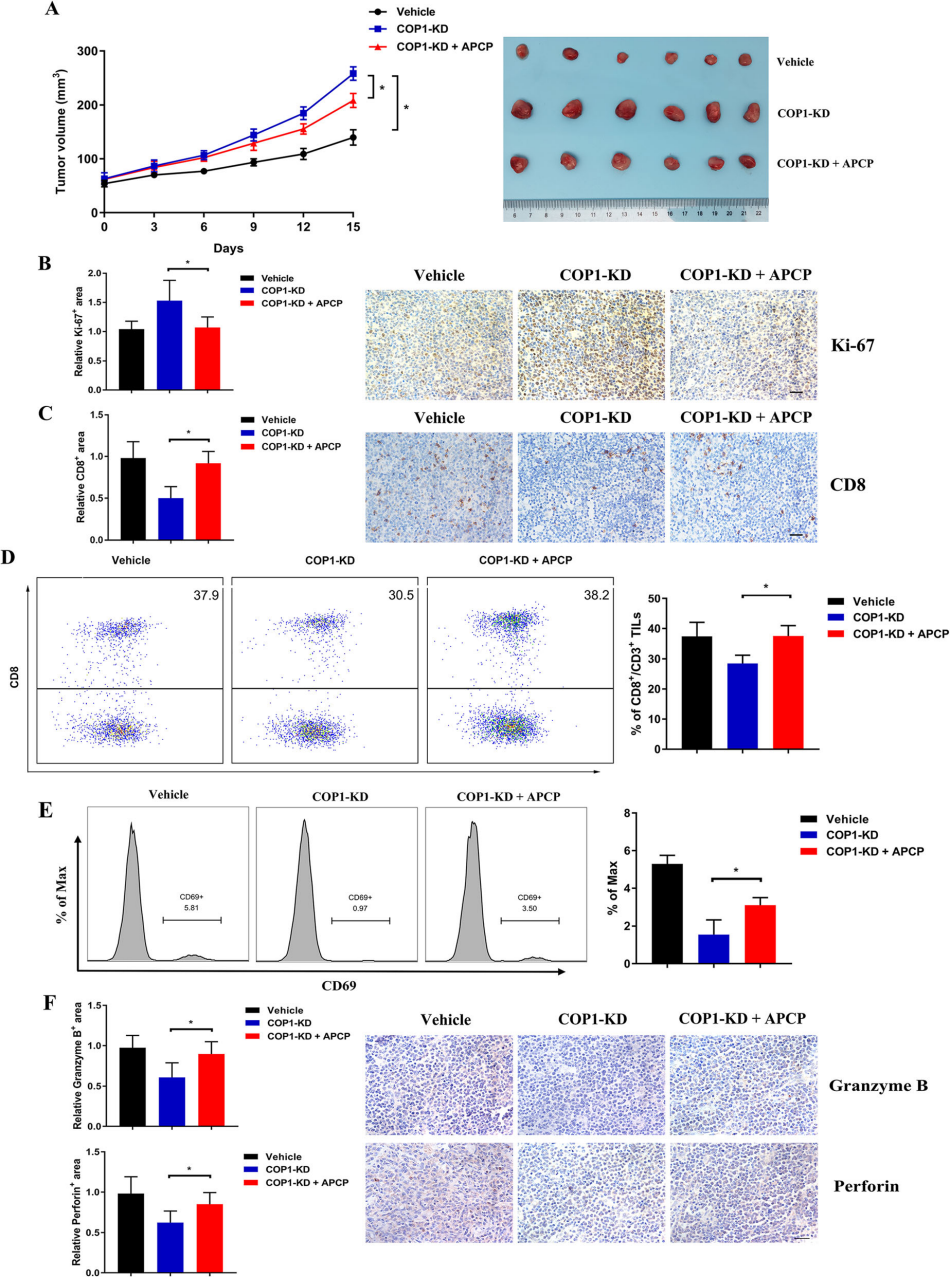
COP1 and APCP exhibited in vivo activity on B-cell lymphoma (**a-b**) APCP treatment significantly decreased the tumor growth rate in COP1 knockdown cells (**a**), consistent with Ki-67 expression in IHC analysis **b**. Statistical tests were performed using tumor volume data on Day 15. **c** IHC analysis showed that fewer CD8^+^ T-TILs caused by COP1 knockdown were significantly rescued by APCP treatment. **d** Representative flow cytometry data and cumulative data for CD8^+^ T-TILs isolated from murine model tumors. In all experiments, cells were first gated on the CD3^+^ population and then on the CD8^+^ population. **e** Representative flow cytometry data and cumulative data for CD69-positive CD8^+^ T-TILs. **f** IHC analysis of granzyme B and perforin Asterisk (*) indicates a significant difference. *, P < 0.05

To further investigate the effects of COP1 and CD73, we constructed murine models with COP1 knockdown or its negative control transfected A20 cells, with or without treatment with the CD73 inhibitor APCP. The increased tumor burden formed by COP1 knockdown cells was significantly abrogated by APCP treatment, which was consistent with our observations of Ki-67 expression (Fig. 9a-b). Additionally, IHC analysis showed a decreased infiltration rate of CD8^+^ T cells with downregulated COP1, which was significantly reversed by APCP (Fig. 9c). Additionally, among CD3^+^ T-TILs, CD8^+^ T-TILs decreased with lower levels of COP1 and were significantly reversed by APCP treatment (Fig. 9d). Functionally, COP1 knockdown decreased the CD69-positive CD8^+^ T-TIL proportion, and the effect was reversed by APCP (Fig. 9e). Cytotoxic molecules (i.e., granzyme B, perforin) was also decreased in CD8^+^ T-TILs with COP1 knockdown and reversed by APCP (Fig. 9f).

### The miR-340-5p/KMT5A axis regulated the cell biology of DLBCL cells

**Fig. 10:**
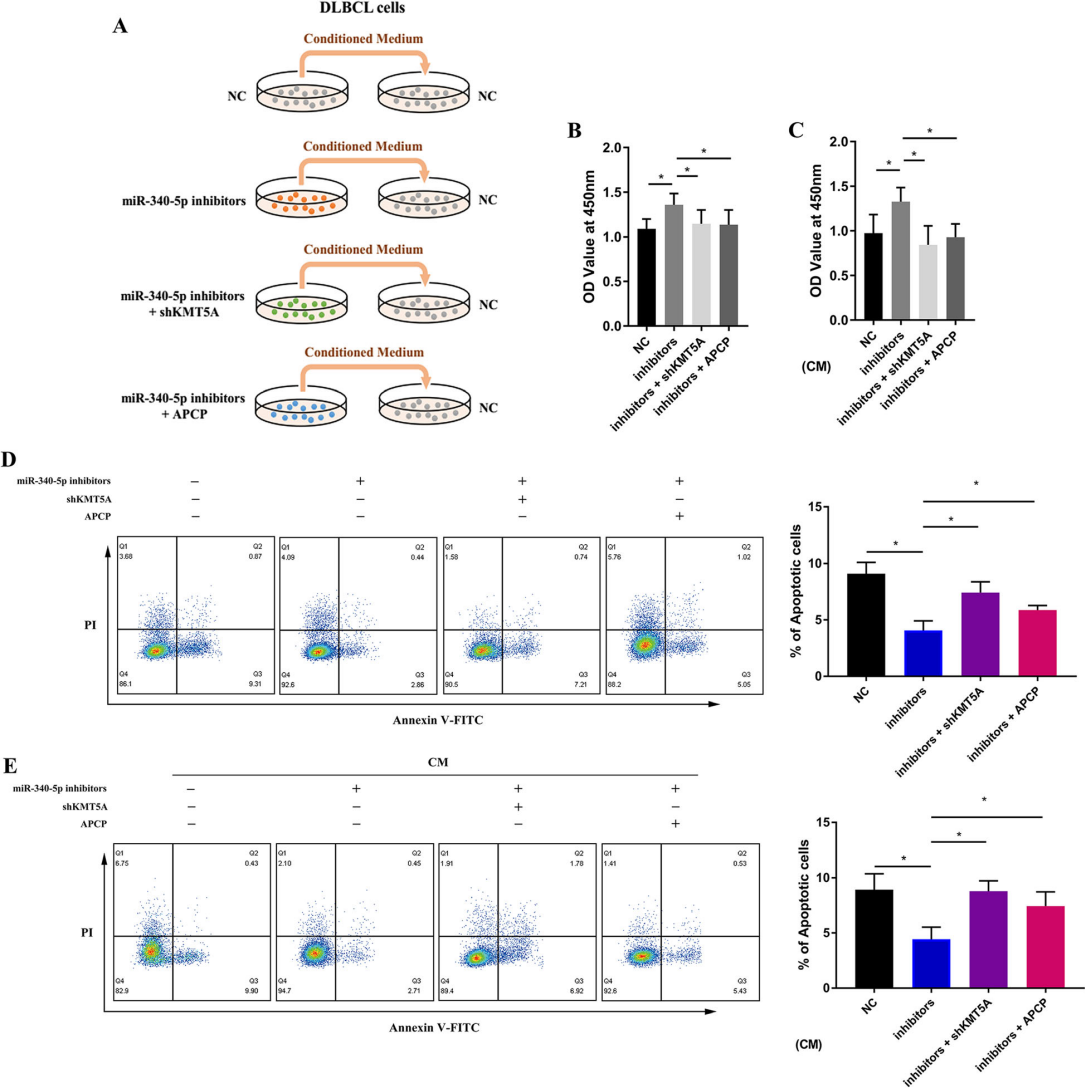
MiR-340-5p affected DLBCL cell biological activity. **a** Schema for DLBCL cells treated with conditioned medium as indicated. **b** DLBCL cell viability was increased by miR-340-5p inhibition and significantly reversed by KMT5A knockdown or APCP. **c** For negative control DLBCL cells, the viability was changed by CM treatment, as indicated. **d** Representative examples and cumulative data of flow cytometry. The DLBCL cell apoptosis rate was decreased by miR-340-5p inhibition and significantly reversed by KMT5A knockdown or APCP. **e** Representative examples and cumulative data of flow cytometry. For negative control DLBCL cells, the apoptosis rate was changed by CM treatment, as indicated. Asterisk (*) indicates a significant difference. *, P < 0.05

In experiments performed in the A20-injected murine model, we observed an influence of Ki-67 expression on miR-340-5p/KMT5A axis regulation. We suggested that the miR-340-5p/KMT5A axis not only facilitated CD8^+^ T-TILs but also inhibited the biological activities of DLBCL cells. Next, to investigate the impact of the miR-340-5p/KMT5A axis on the biological activities of DLBCL cells, we established LY-1 cell lines that stably transfected shKMT5A or with APCP treatment, with or without miR-340-5p inhibitor (Fig. 10a). Moreover, since the miR-340-5p/KMT5A axis regulated extracellular components, we substituted the culture medium of negative control (NC) cells with the supernatant of the above described cells as conditioned medium (CM) (Fig. 10a). miR-340-5p inhibition significantly promoted tumor cell viability, and KMT5A knockdown or APCP significantly reversed this impact (Fig. 10b). After CM substitution, similar results were observed in NC cells cultured with CM as indicated (Fig. 10c). Consistently, miR-340-5p inhibition significantly inhibited the DLBCL apoptosis rate compared with that of negative control cells, which was reversed by KMT5A knockdown or APCP (Fig. 10d). Similar apoptotic effects on DLBCL cells were observed after CM derived from differently treated tumor cells was added (Fig. 10e). Our results demonstrated that the miR-340-5p/KMT5A axis regulated the biological activities of DLBCL cells via extracellular components.

## Discussion

DLBCL is an aggressive heterogeneous malignancy with different biology and poor clinical outcomes. Recently, immunotherapies including immune checkpoint inhibitors and CD19 chimeric antigen receptor T cells (CAR-T cells) have guided novel clinical treatment strategies. Given that aberrant miRNA expression in DLBCL and immune reactions can influence the systemic response to immunotherapy, more accessible and effective miRNAs are considered biomarkers and therapeutic targets [36]. Furthermore, cell infiltration and human cancer composition are complex and involve various immune cells and stromal cells in the TME, where T-TILs offer a rich source of CD8^+^ T cells that monitor for and eliminate tumor cells. Although miRNAs and TME play important roles in DLBCL immune reactions, few studies have been conducted to investigate the correlation between them.

In this study, we identified miR-340-5p/KMT5A as CD8^+^ T-TIL- and survival-related molecules by analyzing the “TCGA-DLBC” project. Then, we confirmed miR-340-5p/KMT5A binding and their effects on CD8^+^ T cells in experimental models. After the interference or forced expression of miR-340-5p/KMT5A, we did not observe a significant difference in CD8^+^ T-cell function alternation between direct and indirect coculture methods. This result implied that extracellular components produced by DLBCL cells were modulated, such as adenosine. Experimentally, COP1 was downregulated with KMT5A overexpression, which impaired CD73 ubiquitination. In addition, independent of immunoregulation, the miR-340-5p/KMT5A axis affected the biological activities of DLBCL cells.

A previous study demonstrated that the miR-340-5p level was correlated with the density of tumor-associated macrophages, which secrete factors that stimulate tumor proliferation, invasion and development [37]. Downregulated miR-340-5p promoted tumor-associated macrophage density in the TME both in vitro and in vivo. Specifically, an increase in tumor-infiltrating macrophages correlated with poor prognosis in DLBCL patients [38]. Furthermore, miR-340-5p was reported to be downregulated and critically involved in tumor suppression in many cancer types [37, 39–41]. Consistent with our results, miR-340-5p regulated DLBCL cell biology and tumor burden in both TME-dependent and TME-independent manners.

It was demonstrated that extracellular adenosine accumulated in the TME was largely produced by CD39/CD73 [26]. CD73-expressing tumor cells negatively regulate the antitumor T-cell response and promote T cell apoptosis [42]. In many cancer types, silencing CD73 inhibited tumor cell proliferation, viability, and cell cycle progression, leading to increased cell apoptosis [43, 44]. In our experiments, regarding the effects on CD8^+^ T cells and DLBCL tumor cells, CM derived from tumor cells with the indicated treatments exerted similar functions as the corresponding tumor cells. These results suggested an important role played by CD73.

During recent years, several T-cell-based adoptive immunotherapies have emerged to stimulate and redirect T-cell functions against tumors [45], and the CD8^+^ T-TIL status was associated with the clinical outcome of DLBCL [46]. Numerous innovative T-cell-based immunotherapy approaches have shown promising results in relapsed or refractory DLBCL patients, leading to a number of ongoing clinical trials. Treatment strategies including PD-1, CTLA-4 and CD19 CAR-T cells have attracted significant attention. To further investigate the clinical application potential of miR-340-5p in DLBCL, it should be determined whether miR-340-5p functions on a specific subtype of CD8^+^ T-TILs or a wide range of CD8^+^ T-TILs. Other tumor-infiltrating immune cells associated with miR-340-5p also deserve more significant attention and investigation, especially immune cells related to CD8^+^ T-TILs, such as regulatory T cells (Tregs) and macrophages.

## Conclusions

In summary, by mining RNA-seq data and bioinformatics analysis, our data showed that the miR-340-5p/KMT5A axis correlated with CD8^+^ T-cell infiltration and function in DLBCL via COP1 and CD73 regulation both in vitro and in vivo. The miR-340-5p/KMT5A axis also exerted an antitumor effect on DLBCL cells independent of immune regulation. We propose that miR-340-5p could be a therapeutic target and part of a new approach for immunotherapy.

## Supplementary Information

**Additional file 1: Table 1.** Sequences of miRNA mimics and inhibitors. **Table 2.** Sequences of siRNA or lentivirus for gene silencing.

**Additional file 2: Supplemental Figure 1.** (**A-C**) MiR-340-5p did not significantly affect cell viability (**A**), activation (**B**) or cytokine production (**C**) directly in CD8^+^ T cells independent of DLBCL cells. *ns, no significance*.

**Additional file 3: Supplemental Figure 2.** (**A**) western blotting confirmed that KMT5A was knocked down by 3 different sequences. (**B**) The mRNA level of CD73 was not affected by KMT5A knockdown. (**C-D**) western blotting (**C**) and RT-PCR (**D**) both revealed unchanged expression levels of CD39 after KMT5A knockdown.

**Additional file 4: Supplemental Figure 3.** CD73 protein levels were not changed with MDM2 or MKRN1 knockdown.

**Additional file 5: Supplemental Figure 4.** No significant decrease in body weight was observed in murine models.

## Data Availability

The datasets generated/analyzed during the current study are available.

## Abbreviations

DLBCL: Diffuse large B cell lymphoma
TILs: Tumor-infiltrating lymphocytes
TME: Tumor microenvironment
TCGA: The Cancer Genome Atlas
PBMCs: Peripheral blood mononuclear cells
IHC: Immunohistochemistry
ISH: In situ hybridization
CCK-8: Cell Counting Kit-8
WGCNA: Weighted correlation network analysis
CM: Conditioned medium
ELISA: Enzyme-linked immunosorbent assay
