# Supplementary material for: MiRNA-340-5p mediates the functional and infiltrative promotion of tumor-infiltrating CD8+ T lymphocytes in human diffuse large B cell lymphoma
Source: J Exp Clin Cancer Res. 2020 Nov 10;39:238. doi: 10.1186/s13046-020-01752-2 (PMC7653890; doi:10.1186/s13046-020-01752-2)
Supplement: Supplementary file 1 — Additional file 1: Table 1. Sequences of miRNA mimics and inhibitors. Table 2. Sequences of siRNA or lentivirus for gene silencing. [file 13046_2020_1752_MOESM1_ESM.docx]

**Supplemental information**

**Table 1** Sequences of miRNA mimics and inhibitors.

| miRNA | Sequence | |
| --- | --- | --- |
|  | **Mimics** | **Inhibitors** |
| Negative control | Sense: 5’-UUCUCCGAACGUGUCACGUTT-3’  Antisense 5’-ACGUGACACGUUCGGAGAATT-3’ | 5’-CAGUACUUUUG  UGUAGUACAA-3’ |
| miR-340-5p | Sense 5’-UUAUAAAGCAAUGAGACUGAUU-3’  Antisense 5’-UCAGUCUCAUUGCUUUAUAAUU-3’ | 5’-AAUCAGUCUCA  UUGCUUUAUAA-3’ |

**Table 2** Sequences of siRNA or lentivirus for gene silencing.

| Target gene | Sequence |
| --- | --- |
| hsa-KMT5A | ^#^1 5’-CCGAGGAACAGAAGATCAAAG-3’  ^#^2 5’-TTGAACAGATGGCCTTATATT-3’  ^#^3 5’-GCCTAGGAAGACTGATCAATC-3’ |
| hsa-COP1 | 5’-GCTGGAGTTACAAAGAAGATT-3’ |
| mmu-COP1 | 5’-CCTTGGTATAACAGCACATTA-3’ |
| hsa-MDM2 | 5’-ATTATCTGGTGAACGACAAAG-3’ |
| hsa-MKRN1 | 5’-CGTATAGTGTAGTGTGCAAGT-3’ |
