# Supplementary figures and images for: MiRNA-340-5p mediates the functional and infiltrative promotion of tumor-infiltrating CD8+ T lymphocytes in human diffuse large B cell lymphoma
Source: J Exp Clin Cancer Res. 2020 Nov 10;39:238. doi: 10.1186/s13046-020-01752-2 (PMC7653890; doi:10.1186/s13046-020-01752-2)

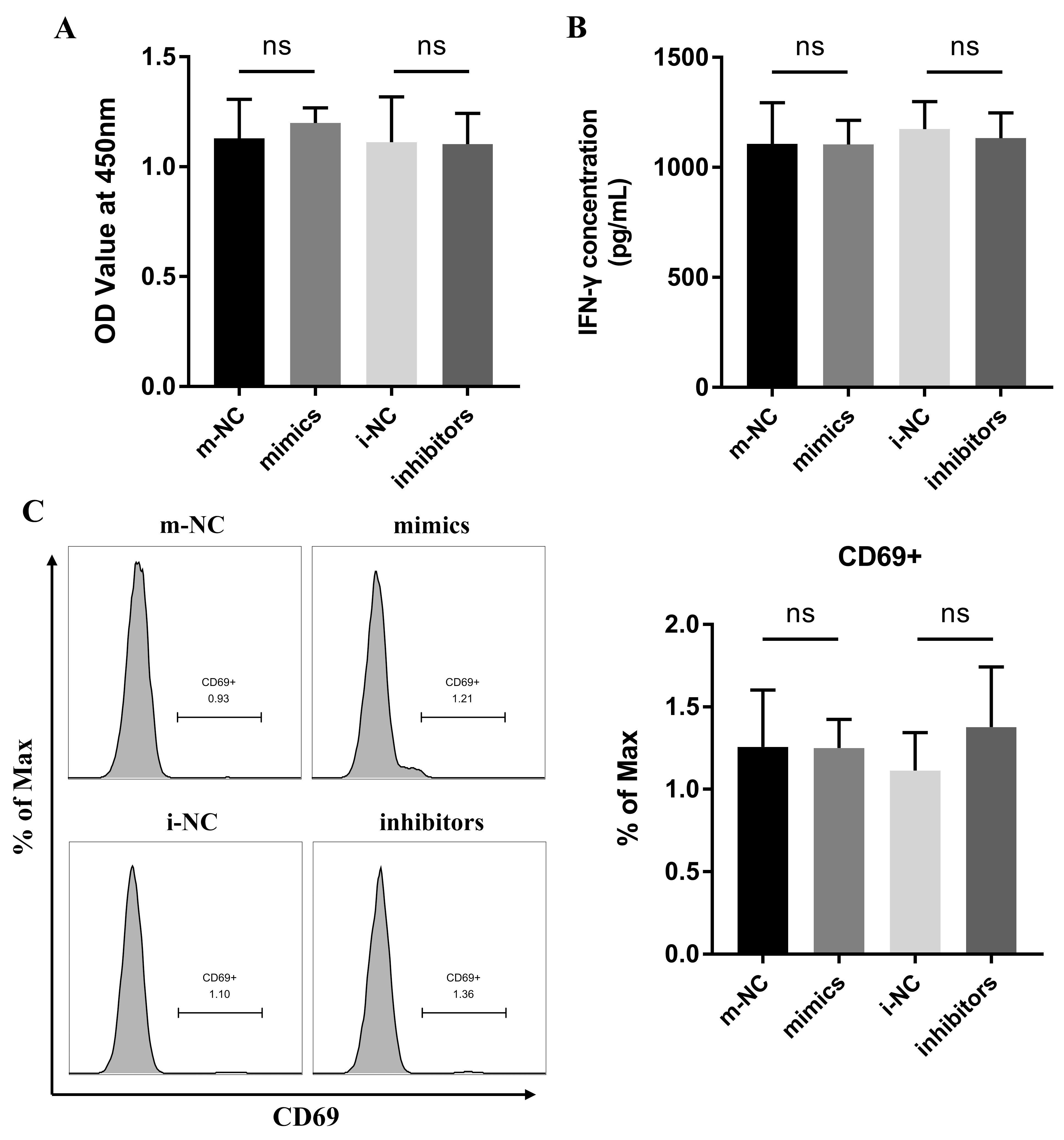

Supplement: Supplementary file 2 — Additional file 2: Supplemental Figure 1. (A-C) MiR-340-5p did not significantly affect cell viability (A), activation (B) or cytokine production (C) directly in CD8+ T cells independent of DLBCL cells. ns, no significance. [file 13046_2020_1752_MOESM2_ESM.tif]

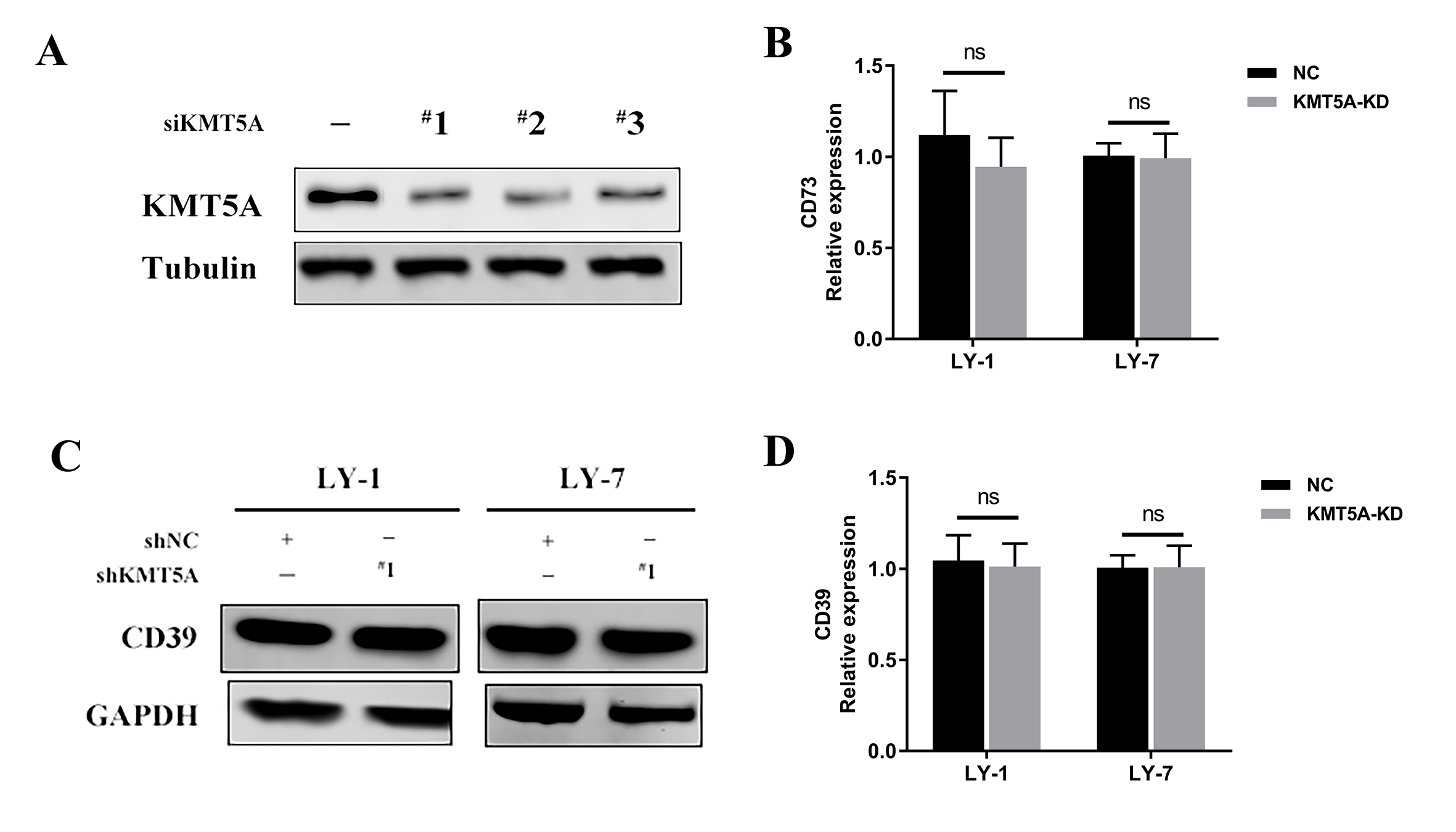

Supplement: Supplementary file 3 — Additional file 3: Supplemental Figure 2. (A) western blotting confirmed that KMT5A was knocked down by 3 different sequences. (B) The mRNA level of CD73 was not affected by KMT5A knockdown. (C-D) western blotting (C) and RT-PCR (D) both revealed unchanged expression levels of CD39 after KMT5A knockdown. [file 13046_2020_1752_MOESM3_ESM.tif]

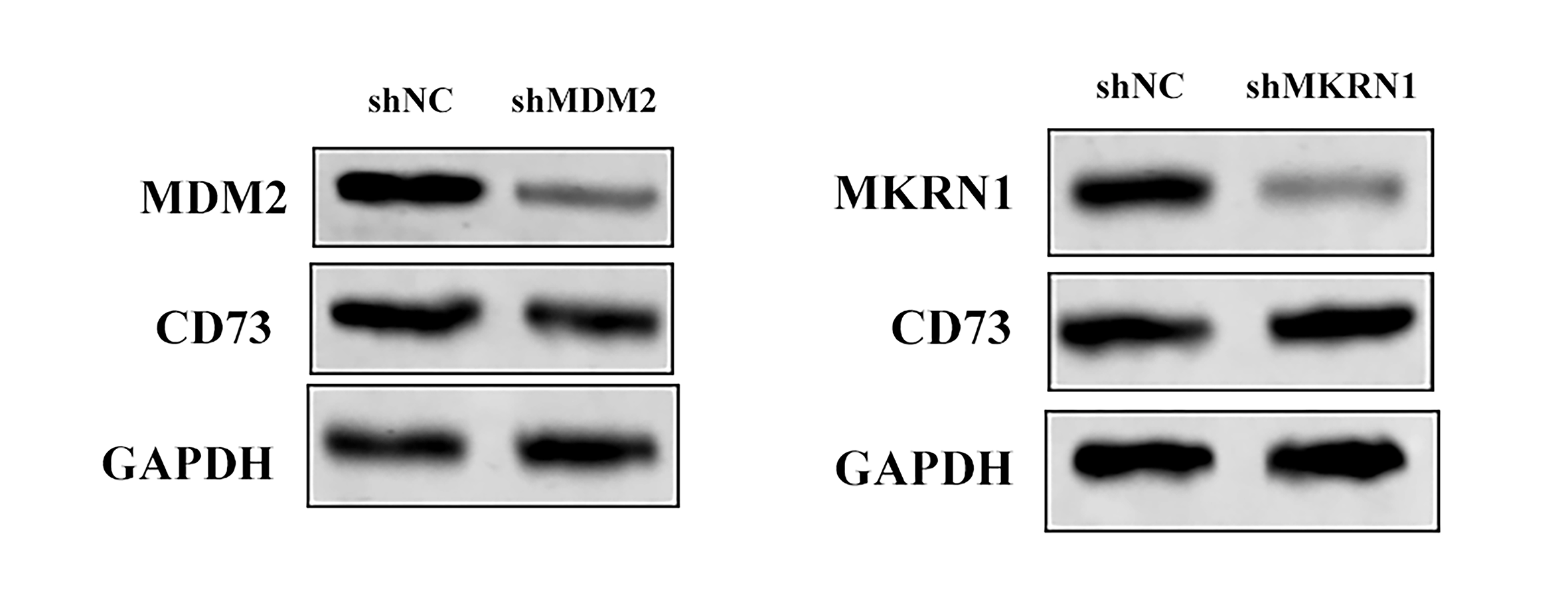

Supplement: Supplementary file 4 — Additional file 4: Supplemental Figure 3. CD73 protein levels were not changed with MDM2 or MKRN1 knockdown. [file 13046_2020_1752_MOESM4_ESM.tif]

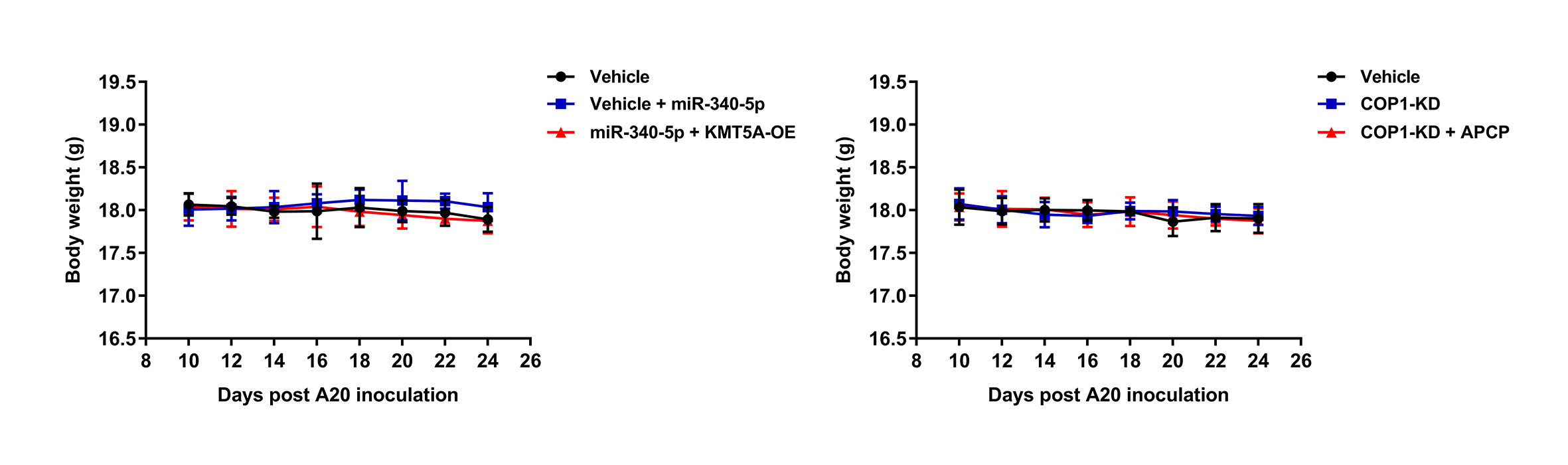

Supplement: Supplementary file 5 — Additional file 5: Supplemental Figure 4. No significant decrease in body weight was observed in murine models. [file 13046_2020_1752_MOESM5_ESM.tif]
